# Anti-Cancer Activity of *Sphaerococcus coronopifolius* Algal Extract: Hopes and Fears of a Possible Alternative Treatment for Canine Mast Cell Tumor

**DOI:** 10.3390/md23120457

**Published:** 2025-11-28

**Authors:** Greta Mucignat, Fatima Lakhdar, Hanane Maghrebi, Ewa Dejnaka, Lorena Lucatello, Bouchra Benhniya, Francesca Capolongo, Samira Etahiri, Marianna Pauletto, Aleksandra Pawlak, Mery Giantin, Mauro Dacasto

**Affiliations:** 1 Department of Comparative Biomedicine and Food Science, University of Padua, 35020 Legnaro, PD, Italy; 2 Laboratory of Marine Biotechnology and Environment, CNRST Labelled Research Unit, Department of Biology, Faculty of Sciences, Chouaib Doukkali University, El Jadida 24000, Morocco; 3 Department of Pharmacology and Toxicology, Faculty of Veterinary Medicine, Wrocław University of Environmental and Life Sciences, 50-375 Wrocław, Poland; 4 Department of Physiology and Pharmacology, University of Georgia, Athens, GA 30602, USA; 5 SMART Pharmacology, Precision One Health Initiative, University of Georgia, Athens, GA 30602, USA

**Keywords:** algal extracts, *Sphaerococcus coronopifolius*, mast cell tumor, RNA-seq, cell cycle, mevalonate pathway

## Abstract

Within the “One Health, One Medicine” and comparative oncology paradigms, algal extracts have attracted attention, containing natural compounds (NCs) with biological activities, including anti-cancer properties. To characterize the biological effects of a *Sphaerococcus coronopifolius* extract (SCE), two canine mastocytoma and two normal cell lines were used. After a preliminary screening of three algal extracts, SCE cytotoxicity was measured using Alamar Blue, Sulforhodamine B, and Neutral Red Uptake assays. After assessing the selectivity versus tumor cells and its chemical characterization, SCE mechanisms of action were investigated using RNA-seq, quantitative PCR, flow cytometry and immunoblotting approaches. SCE showed an IC_50_ comprised between 25 and 35 μg/mL in tumor cell lines, but it also affected normal ones (selectivity index < 2.0). RNA-seq and flow cytometry revealed that SCE negatively affected cell cycle and mevalonate pathway in tumor cells. Additional flow cytometry and immunoblotting investigations suggested a concentration- and time-dependent pro-apoptotic effect of SCE and DNA damage events. In conclusion, SCE demonstrated promising anti-cancer activity in mastocytoma cell lines by targeting the mevalonate pathway, arresting the cell cycle, and inducing apoptosis and DNA damage. Furthermore, the results presented here reinforce the idea that NCs may be promising candidates in comparative anti-cancer chemotherapy.

## 1. Introduction

The green pharmacology approach in veterinary oncology is continually evolving, with natural compounds (NCs) becoming increasingly popular in canine cancer treatment. Overall, such growing interest in NCs research is driven by the goal of discovering new anti-cancer drugs offering fewer side effects. To strengthen the concept, an increasing number of drugs, including anti-tumor agents, derived from natural sources, are being intensively researched [1]. Despite the increasing interest in natural anti-cancer compounds, the understanding of their effects on different tumors of pets (predominantly dogs and cats) is still limited, as most of the preclinical cancer research historically focuses on human or rodent tumor cell lines [2]. However, recent studies have highlighted the potential of NCs in treating canine tumors, demonstrating promising and encouraging anti-cancer activities across various canine tumor cell lines. Active compounds such as toosedanin (from *Melia toosendan*) or artemisinin (from *Artemisia annua*) have shown anti-cancer activity on canine mammary tumor and osteosarcoma cell lines, respectively [3,4].

The advantage of using NC extracts for cancer treatment lies in the potential synergy of multiple compounds within a single extract, where the leading molecule may target one or more biological pathways, while other compounds may affect additional targets or absorption kinetics [5]. In this respect, some studies have already investigated the possible synergistic effect of NC extracts or their active compounds with cytostatic drugs like doxorubicin, cisplatin, toceranib, and carboplatin; moreover, the effects of the combination of diverse extracts have been assessed in different canine cancers in vitro models, too [6,7,8,9]. Overall, these evidences underscore the therapeutic potential of NCs in veterinary oncology, offering alternative and complementary options to traditional anti-cancer chemotherapy protocols.

Mast cell tumor (MCT) is one of the most frequently occurring canine cancer, representing up to 11% of all cutaneous neoplasms. Owing to its prevalence and variable biological behavior, MCT poses a considerable challenge in veterinary oncology. Even the standard therapeutic options, i.e., surgery, radiotherapy and chemotherapy (including the use of tyrosine kinase inhibitors), could show limitations and side effects [10].

There is existing literature indicating how human mastocytosis shows similarities with canine MCT, such as activating *KIT* mutations; this would suggest canines as a good translational animal model for humans [11], strengthening the concept that a multidisciplinary approach based on the emerging “One Health, One Medicine” paradigm could accelerate the development of novel therapies while enhancing our comparative understanding of cancer biology [12].

In response to challenges posed by anti-cancer conventional treatments, a growing interest in exploring NCs, such as algal extracts, as alternative therapies has been recorded [13]. Algae contain a wide range of bioactive compounds with antioxidant, anti-cancer, and anti-inflammatory properties, and hold promise in various medical applications, offering new avenues for treatment, complementing or enhancing the efficacy of existing conventional therapies. However, mechanisms controlling the possible anti-cancer activity of algal bioactive compounds are still unclear and related clinical studies are scarce [13,14].

*Sphaerococcus coronopifolius* (Phylum Rhodophyta; SC) is a red alga widely distributed in the East Atlantic, from Ireland and Great Britain to Canary Islands, as well as in the Mediterranean and Black Seas [15]. It is a rich source of brominated cyclic diterpenes with different chemical structures. Interestingly, several SC bromoditerpenes exhibit a promising anti-tumor activity, as they are cytotoxic in various in vitro models such as HepG2 cells [16]. However, molecular mechanisms underlying the SC cytotoxic effects have not yet been fully characterized, thus highlighting the need for additional research to better understand the possible usefulness of its bioactive compounds.

This study investigated the possible anti-cancer activity of a SC extract (SCE), preliminarily showing greater cytotoxic effects compared to other algal extracts, on an established canine MCT cell line (C2). Upon completion of a more robust cytotoxicity study comprising three different assays, we assessed the selectivity of SCE for tumor cell lines by comparing its cytotoxicity on C2 cells with that obtained on another canine MCT (NI-1) and two non-tumor-derived cell lines, i.e., Cf2Th (from fetal thymus) and Madin-Darby canine kidney (MDCK). Then, a transcriptomic approach (RNA-seq) was applied to deeply understand the transcriptional changes and signaling pathways modulated by the SCE, followed by targeted quantitative RT-PCR (qPCR) confirmatory assays. Finally, SCE effects on cell cycle regulation and apoptosis were investigated by flow cytometry and immunoblotting approaches.

Our results enhance the understanding of biomolecular mechanisms involved in SCE anti-cancer activity, to a wider extent, providing new insights and highlighting possible hopes and challenges to be faced in the use of NCs, and particularly of algal extracts, in comparative anti-cancer chemotherapy.

## 2. Results

### 2.1. Preliminary Cytotoxicity Screening of Algae Extracts

The preliminary cytotoxicity screening for the three algae extracts, derived from SC, *Halopitys incurvus*, and *Laminaria ochroleuca*, was conducted on C2 cells using the Alamar Blue (AB) assay. SCE was the most promising extract, with the lowest half maximal inhibitory concentration (IC_50_: 29.7 μg/mL) among the tested extracts (Figure S1).

### 2.2. SCE Cytotoxicity

**Figure 1:**
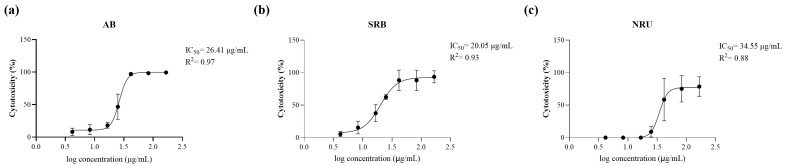
SCE cytotoxicity. Dose–response curves, obtained in C2 cells treated with SCE with three different cytotoxicity tests, i.e., (**a**) AB, (**b**) SRB, and (**c**) NRU assays. For each test, the corresponding IC_50_ and R^2^ values are reported. Each curve is based on three independent biological replicates, with each condition tested in sextuplicate. AB, Alamar blue; IC_50_, half maximal inhibitory concentration; NRU, neutral red uptake; SCE, *Sphaerococcus coronopifolius* extract; SRB, Sulforhodamine B.

A wider cytotoxicity testing, encompassing AB, Sulforhodamine B (SRB), and Neutral Red Uptake (NRU) assays, corroborated the highest cytotoxicity of SCE against C2 cells. Worth noting, the obtained IC_50_ values were in close agreement across different methods: 26.41 μg/mL (AB; R^2^ = 0.97), 20.05 μg/mL (SRB; R^2^ = 0.93), and 34.55 μg/mL (NRU; R^2^ = 0.87; Figure 1).

### 2.3. Selective SCE Cytotoxicity Against Tumor Cell Lines

The selective cytotoxicity of SCE against tumor cell lines was assessed by comparing the IC_50_ values obtained with the AB assay in four cell lines: C2 and NI-1 (MCT) vs. Cf2Th and MDCK (normal cells). Lower IC_50_ values were obtained for C2 and NI-1 cells compared to Cf2Th and MDCK cells (Table S1). This would suggest that SCE exhibits a more pronounced cytotoxicity towards tumor cell lines compared to normal ones. However, the selectivity index (SI; IC_50_ of normal cells/IC_50_ of tumor cells ratio) did not reach a value ≥ 2.0 (which would indicate at least twice higher cytotoxicity on tumor cell lines; see Table S2).

### 2.4. Chemical Characterization of SCE

The chemical characterization of SCE, aimed at identifying compounds potentially responsible for its bioactivity, encompassed Attenuated Total Reflection Fourier Transform Infrared Spectroscopy (ATR-FTIR; Figure S2) for the initial identification of the extract’s functional groups, followed by gas chromatography coupled to mass spectrometry (GC/MS) and high-performance liquid chromatography coupled with mass spectrometry (HPLC/UV/MS; Figure S3) analyses. Based on retention times (Rt) and molecular weights inferred from the GC/MS chromatogram and confirmed by HPLC/UV/MS analysis, seventeen compounds were identified (Table S3). The extract was characterized by the presence of fatty acids, sesquiterpenes, diterpenes, triterpenes, halogenated aromatic compounds, phenolic compounds, steroids, alcans, and organic nitroaromatic compounds. Pentadecanoic acid, methyl ester, and betulin (BET) were identified in SCE after transesterification. Major details about SCE chemical characterization are reported in the Supplementary Files (see Chemical Characterization of SCE).

### 2.5. Differential Gene Expression (DGE) and Functional Analyses

For the subsequent RNA-seq analyses, based on cytotoxicity results previously obtained with AB assay, cells were exposed for 48 h to 8.33 and 16.66 µg/mL of SCE (SCE8 and SCE17), which corresponded to 1/3 and 2/3 of the obtained IC_50_ value, respectively; cells exposed to 0.33% dimethyl sulphoxide (DMSO) were used as control (CTRL). The use of sub-cytotoxic concentrations was aimed at preventing potential confounding effects from extensive cell death, which could obscure the transcriptional regulation of mechanisms occurring before the more obvious phenotypic effects.

Sequencing and alignment results are collected in Table S4, while the complete output of the DGE analysis is available in Table S5.

**Figure 2:**
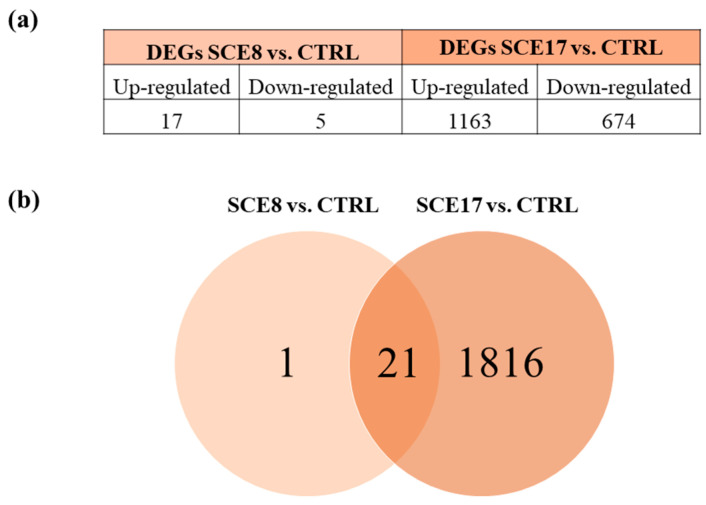
RNA-seq analysis. (**a**) Number of up- and down-regulated DEGs obtained comparing SCE8 vs. CTRL and SCE17 vs. CTRL. (**b**) Venn diagram of the shared DEGs in SCE8 vs. CTRL and SCE17 vs. CTRL comparisons. CTRL, control; DEGs, differentially expressed genes; SCE8, *Sphaerococcus coronopifolius* extract, 8.33 µg/mL; SCE17, *Sphaerococcus coronopifolius* extract, 16.66 µg/mL.

The number of up- and down-regulated differentially expressed genes (DEGs) for each condition, together with the Venn diagram of shared DEGs, is reported in Figure 2. As shown in the Venn diagram, most of the genes found dysregulated following the exposure to the lowest SCE concentration were likewise affected by the highest one, thus suggesting a possible concentration-dependent effect. The expression profile of the functionally annotated DEGs in common between the two comparisons (Figure S4) confirmed this concentration-dependent pattern. The list of these common genes can be found in Table S5. Among them, Hexokinase 2 (*HK2*), Carnitine Palmitoyltransferase 1A (*CPT1A*), Acyloxyacyl Hydrolase (*AOAH*), Pim-2 Proto-Oncogene (*PIM2*), and Laminin Subunit Gamma 2 (*LAMC2*) are worth mentioning.

Given the substantial overlap between DEGs identified at both concentrations, and considering that the highest dose appeared to elicit a broader and more pronounced transcriptional response, the functional enrichment analysis was performed on the up- and down-regulated DEGs modulated by the higher dose. In this respect, the Gene Ontology (GO) over-representation analysis (Table S6) performed on the DEGs obtained from SCE17 vs. CTRL comparison showed 46 up-regulated terms and 49 down-regulated. As for Kyoto Encyclopedia of Genes and Genomes (KEGG) enrichment analysis (Table S6) 15 and 13 pathways resulted up- and down-regulated, respectively.

Considering the GO biological processes enriched by down-regulated genes, most of them were associated with cell cycle and cell division (GO:0007049; GO:0051301; GO:0010564). Chromosome segregation and organization, as well as nuclear division, were also enriched (GO:0007059; GO:0051276; GO:0051985; GO:0000280; Figure 3a). Among the genes belonging to these pathways, we can mention Polo-like kinase 1 (*PLK1*), Cyclins A2 (*CCNA2*), B1 (*CCNB1*), B2 (*CCNB2*), and B3 (*CCNB3*), Aurora Kinase A (*AURKA*) and B (*AURKB*), RAD51 Recombinase (*RAD51*), H2A.X Variant Histone (*H2AX*), NDC80 Kinetochore Complex Component (*NDC80*), and Cell Division Cycle 20 and 25A (*CDC20* and *CDC25A*). The cholesterol synthesis pathway was likewise differentially regulated; for example, the sterol and secondary alcohol biosynthetic and metabolic processes were negatively enriched (GO:0016125; GO:0016126; GO:1902653; GO:1902652). Among the noteworthy genes, we can include Squalene Epoxidase (*SQLE*), 24-Dehydrocholesterol Reductase (*DHCR24*), 3-Hydroxy-3-Methylglutaryl-CoA Synthase 1 (*HMGCS1*), Sterol Regulatory Element Binding Transcription Factor 1 (*SREBF1*), 7-Dehydrocholesterol Reductase (*DHCR7*), Mevalonate Kinase (*MVK*), and NAD(P) Dependent Steroid Dehydrogenase-Like (*NSDHL*). Additional DEGs negatively modulated and possibly related to these same biological processes were Acetyl-CoA Acetyltransferase 2 (*ACAT2*), 3-Hydroxy-3-Methylglutaryl-CoA Reductase (*HMGCR*), Cytochrome P450 Family 51 Subfamily A Member 1 (*CYP51A*), Mevalonate Diphosphate Decarboxylase (*MVD*), and *HK2*.

**Figure 3:**
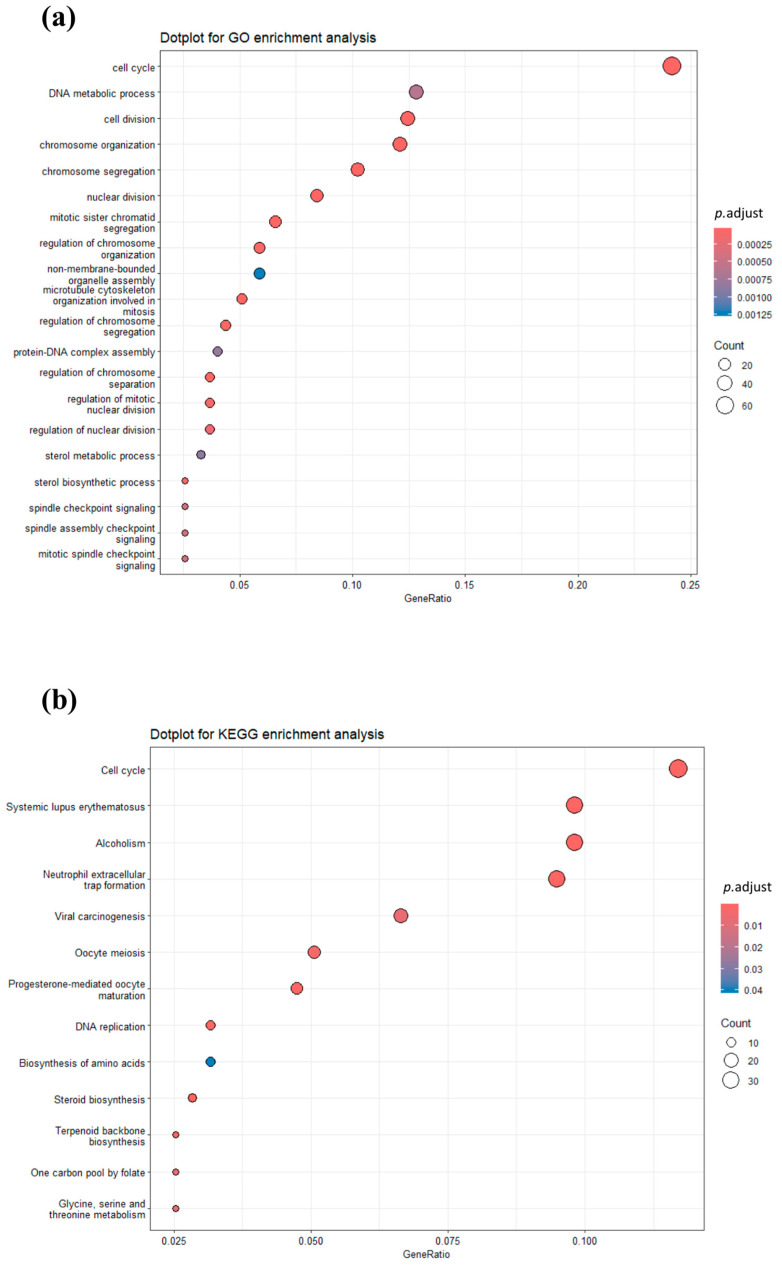
GO and KEGG analysis. (**a**) Dot plots of the 20 most significant GO terms and (**b**) KEGG pathways enriched by down-regulated DEGs (SCE17 vs. CTRL). The dot size represents the number of genes belonging to each pathway. The color gradient is related to the level of significance, adjusted with the Benjamini–Hochberg (BH) method. BH, Benjamini–Hochberg; CTRL, control; GO, Gene Ontology; KEGG, Kyoto Encyclopedia of Genes and Genomes; DEGs, differentially expressed genes; SCE17, *Sphaerococcus coronopifolius* extract, 16.66 µg/mL.

The KEGG enrichment analysis results corroborated the negative modulation of cell cycle and steroid biosynthesis pathways (Figure 3b), as shown by the down-regulation of “Cell cycle” (cfa04110), “DNA replication” (cfa03030), “Steroid biosynthesis” (cfa00100), and “Terpenoid backbone biosynthesis” (cfa00900) biological processes in SCE17-exposed cells.

If we consider only GO terms enriched by the up-regulated genes, worth of mention are the immune system (GO:0050900; GO:0002376; GO:0001816; GO:0050729; GO:0006935), angiogenesis (GO:0045765; GO:1901342) and lipid metabolism (GO:0006629) related ones (Figure S5). Key genes involved in these pathways comprise CXC Motif Chemokine Ligand 13 (*CXCL13*), Tumor Necrosis Factor (*TNF*), Fms Related Receptor Tyrosine Kinase 1 (*FLT1*), Interleukin 18 (*IL18*), C-X-C Motif Chemokine Receptor 2 (*CXCR2*), *AOAH*, and *CPT1A*.

With regard to KEGG enrichment analysis (Figure S6), cancer-related terms like “Pathways in cancer” (cfa05200), “PI3K-Akt signaling pathway” (cfa04151), “MAPK signaling pathway” (cfa04010), and “JAK-STAT signaling pathway” (cfa04630) were positively enriched, too.

Among the DEGs positively modulated in SCE17-exposed cells, we can highlight genes involved in cancer progression, such as *FOS*, *JUN*, and *JUNB* proto-oncogenes, Cyclin Dependent Kinase Inhibitor 1A (*CDKN1A*), and Tumor Protein 53 Inducible Nuclear Protein 1 (*TP53INP1*).

### 2.6. Confirmatory Assays

To validate transcriptomic data, the mRNA expression of 9 target DEGs was determined through qPCR assays using mRNA isolated from C2 cells exposed to SCE17. For most of the target DEGs, the qPCR confirmatory assays outputs were consistent with transcriptomic data, as shown by the Spearman correlation factor (*r* = 0.98; *p* < 0.0001) and the similarity in the magnitude of response (fold changes, fc), as observed in cells exposed to SCE17 (Figure S7).

The same DEGs and related qPCR assays were used to highlight and confirm the selective cytotoxicity of SCE against tumor cell lines, following the incubation of C2, NI-1 (MCT), Cf2Th, and MDCK (normal cell lines) for 48 h with SCE17. Briefly, NI-1 cells showed target gene expression profiles comparable to those obtained with C2 cells. However, Cf2Th cells showed minimal changes and/or opposite trends compared to MCT cell lines. Worth mentioning, MDCK cells showed, to a certain extent, a response comparable to that of NI-1 and C2 cells (Figures S8 and S9). A more detailed description of confirmatory qPCR results of SCE selectivity against cancer cells is reported in the Supplementary Files (see Confirmatory qPCR results of SCE selectivity against tumor cell lines) associated with this article.

**Figure 4:**
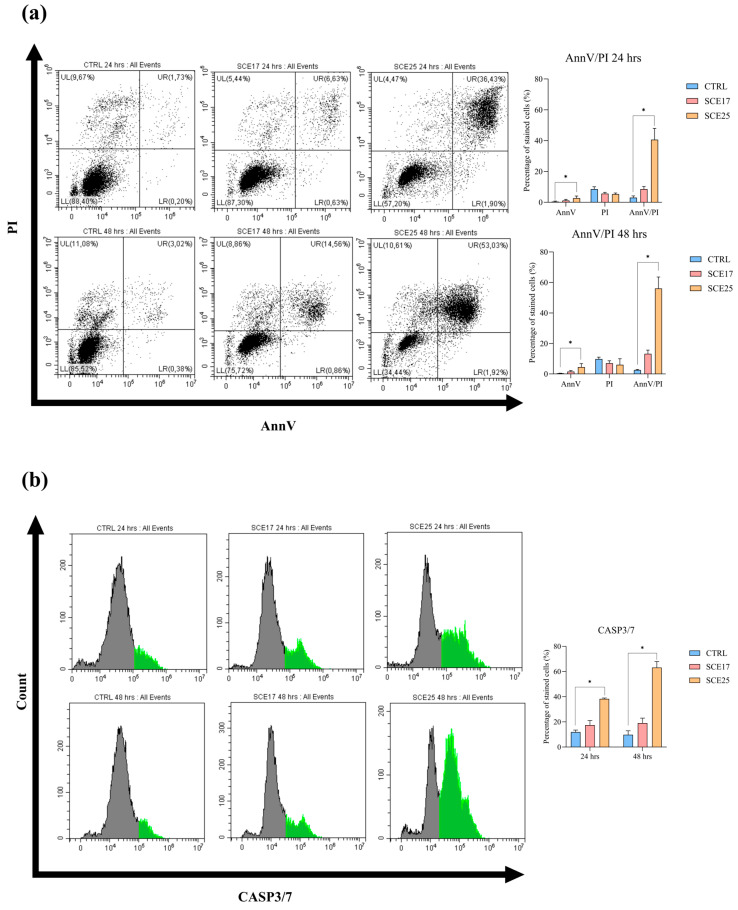
Flow cytometry results for AnnV/PI and CASP3/7. C2 cells were exposed to SCE17 and SCE25 for 24 and 48 h. Panel (**a**) shows the percentage of AnnV, PI, and AnnV/PI positive cells, while panel (**b**) reports the results related to the activity of CASP3/7 in all the conditions tested. Graphs presenting the statistical analysis are reported alongside explanatory dot plots (**a**) and histograms in (**b**). In the dot plots (**a**), the population is divided according to the positivity for either AnnV (lower-right quadrant, LR), PI (upper-left quadrant, UL), both (upper-right quadrant, UR), or neither (lower-left quadrant, LL). In the histograms (**b**), the population of cells in which CASP3/7 is active is highlighted in green. For all the experiments, three biological replicates were considered for each condition. Statistical analysis: Kruskal–Wallis test followed by Dunn’s multiple comparison test. *: *p* < 0.05. AnnV, Annexin V; AnnV/PI, Annexin V/Propidium iodide; CASP3/7, Caspase 3/7; CTRL, control; PI, Propidium iodide; SCE17, *Sphaerococcus coronopifolius* extract, 16.66 µg/mL; SCE25, *Sphaerococcus coronopifolius* extract, 25 µg/mL.

**Figure 5:**
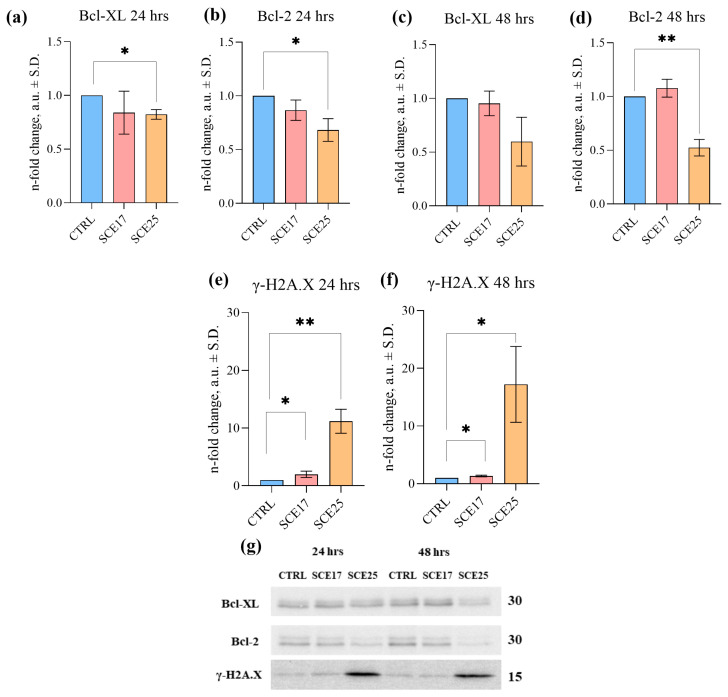
Immunoblotting results for Bcl-2, Bcl-XL, and γ-H2A.X. C2 cells were exposed to SCE17 and SCE25 for 24 and 48 h. Panels (**a**,**b**,**e**) show Bcl-2 (**a**), Bcl-XL (**b**) and γ-H2A.X (**c**) expression after 24 h of exposure. The results collected after 48 h of treatment are reported in panels (**c**,**d**,**f**). In panel (**g**), representative bands for immunoblotting investigations are reported. Results were normalized to the total amount of protein in each lane (total protein normalization). Three biological replicates were considered for Bcl-2 and Bcl-XL, while four were used for γ-H2A.X. Statistical analysis: one-sample *t*-test. *: *p* < 0.05; **: *p* < 0.01. Bcl-2, B-cell lymphoma 2; Bcl-XL, B-cell lymphoma-extra-large; CASP3/7; CTRL, control; SCE17, *Sphaerococcus coronopifolius* extract, 16.66 µg/mL; SCE25, *Sphaerococcus coronopifolius* extract, 25 µg/mL.

Given the strong representation of cell cycle-related genes and terms in the RNA-seq results, flow cytometry and immunoblotting assays were used for validation purposes. Propidium iodide was used to confirm the regulation of cell cycle progression, while different markers were considered to assess the induction of apoptosis and DNA damage. To do so, cells were incubated with both SCE17 and a higher concentration corresponding to the SCE IC_50_ value (25 µg/mL; SCE25). The highest SC concentration (SCE25) caused a significant (*p* < 0.05) increase in Annexin V (AnnV, i.e., early apoptotic) and AnnV/propidium iodide (PI; late apoptotic) positive cells at both 24 and 48 h post-exposure (Figure 4a); a similar significant increase (*p* < 0.05) was observed in caspase 3/7 (CASP3/7) activated cells, too (Figure 4b). Overall, SCE25 also significantly (*p* < 0.05) decreased B-cell lymphoma 2 and extra-large (Bcl-2 and Bcl-XL) protein expression. This decrease was statistically significant at 24 h for both proteins, while at 48 h only for Bcl-2 (Figure 5a–d,g).

**Figure 6:**
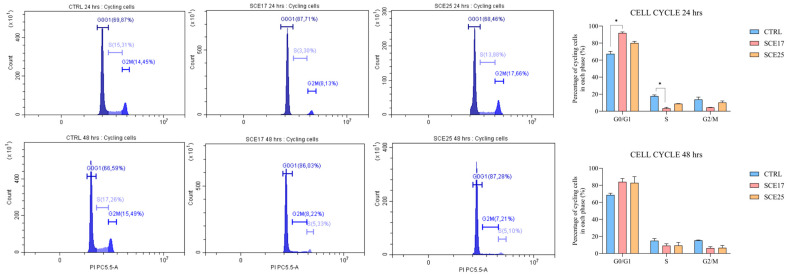
Cell cycle analysis. C2 cells were exposed to SCE17 and SCE25 for 24 and 48 h. The percentage of cycling cells in each phase is reported for each condition tested. Graphs reporting the statistical analysis are shown alongside explanatory histograms. For all experiments, three biological replicates were used per condition. Statistical analysis: Kruskal–Wallis test followed by Dunn’s multiple comparison test. *: *p* < 0.05. CTRL, control; SCE17, *Sphaerococcus coronopifolius* extract, 16.66 µg/mL; SCE25, *Sphaerococcus coronopifolius* extract, 25 µg/mL.

Regarding the cell cycle analysis, after 24 h of incubation, SCE17 significantly increased the number of cells in the G0/G1 phase (*p* < 0.05), and decreased the number of cells in the S phase (*p* < 0.05; Figure 6). However, SCE25 significantly increased the number of cells in SubG0 at both 24 and 48 h post-exposure (*p* < 0.05; Figure S10).

Finally, to assess the effects of SCE on DNA damage, the expression of the γ-H2A.X protein was investigated by immunoblotting. A significant increase in phosphorylated H2A.X was observed in cells exposed to SCE17 and SCE25 at both time points (Figure 5e–g).

## 3. Discussion

Cancer is considered one of the foremost public health challenges worldwide, with its impact escalating in recent decades [17]. It is responsible for 30.3% of premature deaths in individuals aged 30 to 69, and is among the top three causes of death in this age group in 177 out of 183 countries [18]. Chemotherapy, in its various forms, is the prevalent approach for cancer management. Chemotherapy targets proliferating neoplastic cells, but its influence on healthy cells and the resulting adverse effects are an important complication [17]. An additional challenge is overcoming cancer therapy resistance, which is often multifactorial, while the presence of undruggable cancer drivers adds more layers of complexity [18]. Addressing cancer chemotherapy side effects necessitates a multidisciplinary strategy and integrative approaches, combining conventional with evidence-based complementary medicines/therapies, nutritional medicine, and lifestyle modifications. In such a context, the growing interest in the use of NCs for cancer treatment is fully justified [1,17,19].

Bioactive NCs are prospective agents in oncological therapy owing to their capacity to target neoplastic cells, acting through potentially relevant mechanisms. These chemicals, sourced from natural entities like marine algae (macroalgae and microalgae), show multiple anti-cancer effects, such as cell cycle arrest, apoptosis induction, angiogenesis suppression, and signaling pathway regulation. In addition, NCs may have improved anti-cancer benefits when used in combination, either with another NC or traditional anti-cancer drugs, via complementary mechanisms (e.g., immunomodulation, apoptosis induction, and angiogenesis suppression [20,21,22]). As a whole, these evidences underscore the potential of NCs in cancer therapy, offering further options to traditional protocols.

When discussing a multidisciplinary approach to cancer therapy, we cannot avoid mentioning the concept of comparative oncology. Spontaneous tumors of pets, and particularly dogs, are a powerful resource for a better understanding of human cancer. Companion dogs are not only affected by some types of cancer occurring in humans [23]. Significant genetic, molecular, and histological analogies between canine and human neoplasms have also recently been highlighted, including the complex mechanisms of drug resistance. In addition, dogs share numerous exposure factors with humans and show similarities in immune and treatment responses [12,23]. Comparative oncology pursues two main objectives: (a) to offer a deeper and more integrative understanding of what cancer is; (b) to find novel options for treatment by leveraging the natural cancer resistance mechanisms observed across various species [24]. Therefore, the “One Health, One Medicine” paradigm could accelerate the development of novel therapies and enhance our comparative understanding of cancer biology [12].

MCT is the most frequently occurring and challenging skin tumor of dogs [10]. Interestingly, existing literature demonstrates molecular similarities between canine MCT and human mastocytosis (e.g., activating *KIT* mutations, a good response to tyrosine kinase inhibitors, and the occurrence of primary or secondary drug resistance); hence, canine MCT would be considered a good translational animal model for humans [11].

Nowadays, increasing interest is attributed to the use of algal extracts for complementing or enhancing the efficacy of existing conventional anti-cancer therapies, although the molecular mechanisms controlling the possible anti-cancer activity of algal NCs remain largely unexplored [13,14]. This is the case of SC. This red alga contains NCs with a promising anti-tumor activity [16]; however, the molecular mechanisms underlying these cytotoxic effects have not yet been fully characterized.

Taking all this evidence as a whole, in the presented study, we characterized the potential anti-cancer activity of a red alga extract (SCE) on an established canine MCT cell line (C2). To this purpose, after a preliminary screening of candidate algae extracts’ effects on cell viability, we (a) executed a more robust cytotoxicity study, (b) assessed SCE selectivity for tumor cell lines, (c) investigated the transcriptional changes and signaling pathways modulated by the SCE, (d) followed by qPCR, flow cytometry, and immunoblotting confirmatory assays.

Algae are known for their richness in NCs with anti-cancer properties [13]. In previous studies, the pharmacological effect of algal extracts on mast cells has been mainly linked to several anti-allergic compounds such as phlorotannins, polysaccharides, carotenoids, phycocyanin, and polyunsaturated fatty acids [25]. In two successive studies, Gueck and collaborators confirmed that three fatty acids (i.e., α-linolenic acid, γ-linolenic acid, and docosahexaenoic acid) affected the production of prostaglandin E and the release of histamine in the C2 cell line [26,27].

Among the three algae extracts we considered in the preliminary cytotoxicity screening with AB assay, SCE proved to be the most effective (lower IC_50_) in reducing C2 cells’ viability. The wider cytotoxicity testing using three different assays corroborated the high SCE cytotoxicity against the C2 cell line. Worth mentioning, the obtained IC_50_ values were similar, below 50 μg/mL, and consistent with other studies conducted on algal extracts from various sources or of different types [28,29].

Cell lines are important tools in anti-cancer drug discovery, and particularly in predicting candidate efficacy and toxicity. Nowadays, ever-increasing value is given to compare the aforementioned effects on cancer and healthy cells [30]. A very common way to assess the potential of a candidate anti-cancer compound (including NCs) is to determine its SI, which is the ratio between its cytotoxicity (IC_50_) in normal compared to tumor cell lines. A common threshold for considering NCs highly selective is an SI value usually greater than 2 [31]. In our experimental conditions, SCE showed a more pronounced cytotoxicity in tumor cell lines (C2 and NI-1) with respect to normal ones (Cf2Th and MDCK). Nevertheless, the SI value turned out to be below 2. In this regard, it should be emphasized that the response of normal cells has already been shown to vary substantially depending on the candidate compound and/or the chosen cell line [32,33]. In addition, to our knowledge, a canine normal mast cell line is not available; moreover, very few normal canine cell lines, isolated from healthy tissues (e.g., MDCK), are available. In our opinion, to overcome this limitation and avoid potential interpretative biases, it would likely be necessary to employ a more comprehensive panel of cell lines with respect to the one we used in our study.

Both SC methanol and dichloromethane extracts have shown high cytotoxic and anti-proliferative effects in HepG2 and Caco-2 cells [34,35]. Further studies on the characterization of SC chemical profile revealed the presence of structurally diverse bromoditerpenes, such as bromosphaerol, sphaerococcenol A, 12S-hydroxy-bromosphaerol, and 12R-hydroxy-bromosphaerol. Overall, these NCs showed a significant anti-tumor activity (i.e., low IC_50_ values) in various tumor cell lines, including both apoptosis-resistant and apoptosis-sensitive human tumor cell lines [16,36,37,38,39,40,41,42,43]. Furthermore, the 12R-hydroxy-bromosphaerol was the most effective NC in more complex living models, such as stem and healthy cells co-cultures mimicking the tumor microenvironment [36].

The chemical characterization of SCE revealed it is a rich source of fatty acids, i.e., tetradecanoic acid methyl ester, pentadecanoic acid methyl ester, phytol, hexadecanedioic acid, dimethyl ester, and (Z)-9-9-octadecenoic acid methyl ester. Intriguingly, SCE turned out to be particularly rich in a triterpene, i.e., BET. This NC has been reported to inhibit proliferation in different tumor cell lines, inducing apoptosis, mitochondrial damage, and cell cycle arrest [44]. Moreover, the BET anti-proliferative and pro-apoptotic potential has also been investigated in three canine tumor cell lines: T-cell lymphoma (CL-1), B-cell lymphoma (CLBL-1), and osteosarcoma (D-17). Overall, BET showed a strong anti-tumor activity, specifically causing an arrest in the S phase for CL-1 and D-17 cells and in the G0/G1 phase for CLBL-1 cells [45].

In the present study, transcriptomic investigations, performed on C2 cells at sub-cytotoxic concentrations (SCE8 and SCE17), showed a prevalent effect on genes involved in the cell cycle. Indeed, many DEGs showed some relationship with the mevalonate pathway (MELPATH), which is indeed intimately related to the cell cycle [46,47]. MELPATH encompasses a cascade of reactions responsible for de novo cholesterol synthesis and the production of many non-sterol isoprenoid derivatives. In our experimental conditions, most of the transcripts related to MELPATH (e.g., *ACAT2*, *HMGCS1*, *HMGCR*), as well as genes involved in the subsequent steps of cholesterol biosynthesis (e.g., *SQLE*, *CYP51A*, *NSDHL*, *DHCR7*), were down-regulated. Recent literature further strengthens the link between the cell cycle and MELPATH. For example, *SREBF1*, the gene coding for srebp1 (i.e., the sterol regulatory element binding protein 1), which is responsible for the transcriptional regulation of sterol biosynthesis enzymes, here down-regulated by SCE, was previously associated with cell cycle arrest in G1 phase [48,49]. Its inhibition, in fact, causes the repression of lipid metabolism, as well as glucose uptake and glycolysis in tumor cells [50]. Interestingly, BET inhibits the expression of srebp1 transcription factor [51]. Another interesting MELPATH gene, known to be targeted by phytochemicals, is *TP53* [52]; its mutational state contributes to the modulation of the expression of several genes involved in this pathway [52,53]. Worth mentioning, the C2 cell line carries a missense homozygous variant in *TP53* DNA binding domain (p.V162A: [54]), while the non-cancerous MDCK cell line shows a wild-type *TP53* [55]. Surprisingly, the second normal cell line tested in the study (Cf2Th) harbors a *TP53* variant (p.C226F) associated with the very low expression of p21 [55,56]. Therefore, we cannot associate with certainty the SCE differential effect to *TP53* mutational status, also because a complete scenario is not yet available; indeed, the mutational status of the NI-1 cell line is still unknown, and the functional characterization of *TP53* variants present in C2 and Cf2Th cells is not yet available [57].

The inhibition of the MELPATH pathway could indicate a more general metabolic reprogramming and a shift from glycolytic to oxidative metabolism. Indeed, glycolysis is the fuel of the MELPATH. This is consistent with *HK2* inhibition and *CPT1A* induction. These genes were already modulated at the lowest extract concentration used (SCE8), suggesting that this could represent an early cellular response to the treatment. The reduction in *HK2* expression could imply an inhibition of glycolysis and, consequently, a decrease in pyruvate and acetyl-CoA availability for the TCA cycle and the MELPATH [48,58,59]. On the other hand, an increased expression of *CPT1A* could be associated with a higher β-oxidation [60].

The inhibition of MELPATH and, more generally, this metabolic shift, could represent the cause of cell cycle arrest. Present RNA-seq data showed a negative regulation of cell cycle progression, and particularly of genes like cyclins (e.g., *CCNA2*, *CCNB1*, *CCNB2*, *CCNB3*), kinases (e.g., *PLK1*, *AURKA*, *AURKB*), and DNA damage-associated genes (e.g., *RAD51*, *H2AX*, *TP53INP1*). The observed effects on cell cycle and MELPATH could be offered as a possible explanation for the relative MDCK sensitivity to SCE. Indeed, many tissues exhibit cell-cycling rates higher than those found in tumors, and this probably represents, at the same time, the limit and the challenging issue of SCE, which targets pathways not fully selective for tumor cell lines [49,61].

In contrast to MELPATH inhibition and cell cycle arrest, KEGG enrichment analysis revealed the activation of the PI3K/Akt pathway at the transcriptional level. However, SCE could directly influence glycolysis, and the regulation of the PI3K/Akt pathway could be seen as an initial attempt to compensate for, and ultimately counteract, a possible apoptotic process.

To investigate more deeply the effects of SCE on the cell cycle and apoptosis, various confirmatory assays were performed. The effect on the cell cycle was more evident after 24 h with SCE17, while the apoptotic effect was more pronounced after 48 h with a higher concentration. Indeed, after 24 h, almost the entire population was in the G0/G1 phase. In addition, higher doses and longer incubation times led to a proportional increase in the number of dead cells (sub-G0 ones). Besides the anti-proliferative effects, SCE also elicited a concentration- and time-dependent pro-apoptotic effect, as demonstrated by significant increases in AnnV/PI positive and CASP3/7 activated cells, as well as the reduced expression, albeit of minor importance, of the anti-apoptotic proteins Bcl-2 and Bcl-XL. Overall, the presented results would confirm the former comparative results obtained with BET on human [62,63] and canine tumor cell lines [45]. In our experimental conditions, we also observed a time- and concentration-dependent increase in γ-H2A.X protein expression that would suggest an additional and subsequent SCE effect on DNA damage. To our knowledge, BET does not seem to be able to activate γ-H2A.X; however, betulinic acid has been shown to induce apoptosis and DNA damage in brain tumor cells by inducing phosphorylation of H2A.X [64]. Fascinatingly, BET can be converted into betulinic acid [65,66]. Studies reporting the direct effect of BET on H2A.X phosphorylation may not have been widely conducted or published, but this does not definitively rule out the possibility. Hence, more in-depth studies are required to clarify this controversial issue. Apart from this, our results suggest that SCE causes a perturbation of the cell metabolism, cell cycle, together with DNA damage and apoptosis.

This study is meant to preliminarily evaluate the potential anti-cancer properties of SCE in a canine mastocytoma model. Crude extracts may offer broader biological activity since they contain a mixture of different molecules rather than a single compound. However, further investigations of individual bioactive compounds could help elucidate the biological activity of SCE and refine its selectivity. It is also important to note that this study was conducted exclusively in vitro. Therefore, preclinical studies in laboratory animals may represent a necessary step to assess the translational opportunities of this research.

## 4. Materials and Methods

### 4.1. Chemicals and Reagents

Cell media Roswell Park Memorial Institute (RPMI) 1640, Dulbecco’s Modified Eagle Medium (DMEM) and Minimum Essential Medium (MEM), fetal bovine serum (FBS), penicillin–streptomycin (P/S), as well as MEM non-essential amino acids (NEAAs) were provided by Gibco (Thermo Fisher Scientific, Waltham, MA, USA). Alanine-glutamine (A/G), sodium pyruvate (PYR), resazurin, sulforhodamine B, and neutral red were supplied by Sigma-Aldrich (Merck kGaA, Darmstadt, Germany). All other consumables were provided by Sarstedt (Nümbrecht, Germany). High-Capacity cDNA Reverse Transcription Kit and qPCR Power SYBR Green PCR Master Mix were distributed by Applied Biosystems (Thermo Fisher Scientific, Rodano, Milan, Italy). Reversible Protein Stain Kit for Nitrocellulose Membranes, Western blot Signal Enhancer, and ECL Western blot Substrate were sourced from Pierce (Thermo Scientific, Rockford, IL, USA). CellEvent Caspase 3/7 Green Flow Cytometry Assay Kit and FxCycle™ PI/RNase Staining Solution were from Invitrogen (Thermo Fisher Scientific, Eugene, OR, USA), while Annexin V-FITC was from Immunostep (Salamanca, Spain). RNeasy mini Kit and RNase-Free DNase Set were from Qiagen (Hilden, Germany).

### 4.2. Algal Sampling

Seaweeds were collected by hand-picking during March and April 2021 from the Sidi Bouzid coast, El Jadida, Morocco (33°16′09″ N, 8°30′–8°45′ W). Three algae were identified from fresh species: two red algae, i.e., SC (Rhodophyceae) and *Halopitys incurvus* (Florideophyceae), and the brown alga *Laminaria ochroleuca* (Phaeophyceae). Algae were thoroughly cleaned to remove epiphytes, sediment, organic debris, and macrofauna. Samples were successively rinsed with seawater, tap water, and distilled water. The rinsed thalli were then completely dried at room temperature and then crushed to a fine powder.

### 4.3. Preparation of Algal Crude Extracts

The three algal extracts were obtained by macerating 50 g of each dried algal powder in a mixture of dichloromethane/methanol (1:1, *v*/*v*) over 72 h. Then, algal extracts were filtered and the solvents removed by rotary evaporation under pressure at 45 °C, until a crude extract was obtained. This one was then stored at 4 °C until use. At that point, algal extracts were dissolved in DMSO to obtain stock solutions to be diluted in culture medium to reach the final required concentrations.

### 4.4. Cell Culture

The canine C2 MCT cell line was kindly provided by Dr. Dubreuil (Centre de Recherche en Cancérologie de Marseille, Marseille, France), while the NI-1 MCT cell line was a generous gift from Prof. Valent (Medizinische Universität, Vienna, Austria) and Drs. Hadzijusufovic and Willmann (Veterinärmedizinische Universität, Vienna, Austria). The non-tumor-derived cell lines Cf2Th and MDCK were purchased from the European Collection of Cell Cultures (Salisbury, UK).

Cell lines were maintained in a humidified incubator at 37 °C with 5% CO_2_. For C2 and NI-1 cells, RPMI-1640 medium supplemented with 10% FBS, 2 mM A/G, 1% P/S, 1% NEAA, and 1 mM PYR was used, while Cf2Th and MDCK cell lines were cultivated in DMEM and MEM, respectively, both supplemented with 10% FBS, 2 mM A/G, 1% P/S, and 1% NEAA.

### 4.5. Preliminary Cytotoxicity Screening of Algal Crude Extracts

A preliminary screening of algal extracts’ effects on cell viability was performed using C2 cells and the Alamar Blue (AB) cytotoxicity test. Full details about cell seeding, treatment conditions, and the AB assay are reported in the Supplementary Files (see Preliminary screening of algal extracts cytotoxicity using AB assay) associated with this article.

### 4.6. SCE Cytotoxicity

Following the results obtained in the aforementioned preliminary screening, which identified SCE as the extract with the lowest half-maximal inhibitory concentration (IC_50_), a more comprehensive assessment of its potential cytotoxicity was evaluated using C2 cells and three different tests: AB, the SRB, and the NRU assays. Likewise to AB assay, the full details of these tests are reported in the Supplementary Files (see SCE cytotoxicity).

### 4.7. Selectivity of SCE Against Tumor Cell Lines

To further substantiate the potential efficacy of SCE, the AB assay was also used to assess the selectivity of SCE for tumor cell lines, basically by comparing its cytotoxicity in C2 cells with another canine MCT (NI-1) and healthy Cf2Th and MDCK cell lines. Apart from the different cell seeding concentrations adopted for Cf2Th and MDCK (1 × 10^4^ cells/well and 1.5 × 10^3^ cells/well, respectively), the treatment conditions and details about the AB assay are those reported in Table S7 and Supplementary Files (see Preliminary screening of algal extracts cytotoxicity using AB assay). Three independent biological replicates, with each concentration tested in sextuplicate, were performed. SI was calculated as described by de Oliveira et al. (2015) [67].

### 4.8. Chemical Characterization of SCE

A fully detailed description of the instruments and methods used for the chemical characterization of SCE is provided in the Supplementary Files (see Chemical characterization of SCE).

### 4.9. Incubation of Cells for Gene Expression Analysis (RNA-seq) and Confirmatory Assays (qPCR, Flow Cytometry, Immunoblotting)

To assess the effects of SCE on the transcriptional profile of C2 cells (RNA-seq) as well as for the execution of qPCR confirmatory assays, the cells were seeded in 6-well flat-bottom plates at a density of 6 × 10^5^ cells/well. Based on cytotoxicity results previously obtained with AB assay (see SCE cytotoxicity), cells were exposed for 48 h to 8.33 and 16.66 µg/mL of SCE; cells exposed to 0.33% DMSO were used as control (CTRL).

Besides C2 cells, additional qPCR confirmatory assays of SCE selectivity against tumor cell lines were executed on NI-1 (MCT) in comparison to Cf2Th (normal thymus) and MDCK (normal kidney) cell lines. To this purpose, NI-1 (6 × 10^5^ cells/well), Cf2Th (2 × 10^5^ cells/well), and MDCK (3 × 10^4^ cells/well) cells were seeded in P6 multi-well plates and exposed only to SCE17 for 48 h.

For confirmatory flow cytometry and immunoblotting investigations, C2 cells were incubated for 24 and 48 h with two SCE concentrations, namely the SCE17 and the SCE25.

Four independent biological replicates were performed for transcriptional and DNA-damage evaluations, while three biological replicates were considered for the other post-translational investigations.

### 4.10. RNA Extraction

At the end of the incubation time, cells in suspension (C2 and NI-1) were pelleted, while medium was aspirated from those growing in monolayer (Cf2Th and MDCK). Cells were at first washed once with PBS containing 0.02% ethylenediaminetetraacetic acid (EDTA), resuspended in 600 μL of RLT buffer (Qiagen, Milan, Italy) containing 6 μL of β-mercaptoethanol, vortexed, and stored at −80 °C until use.

Total RNA was extracted using the RNeasy Mini Kit (Qiagen, Milan, Italy) and quantified as previously reported [68]. All samples intended for sequencing had an RNA Integrity Number (RIN) value > 7 (4150 TapeStation System, Agilent Technologies, Santa Clara, CA, USA).

### 4.11. RNA-Seq Library Preparation and Sequencing

Library preparation and sequencing were performed by Novogene Biotechnology (Cambridge, UK). A total of 12 tagged RNA-seq libraries were prepared and sequenced using a 150 bp no-stranded specific paired-end strategy in an Illumina Novaseq 6000.

### 4.12. DGE Analysis and Functional Analysis

Raw reads underwent quality control and trimming of adapter sequences before the pseudoalignment to the reference transcriptome (ROS_Cfam_1.0, Ensembl release 109) with Kallisto (v.0.48.0). For DGE analysis and functional analysis, edgeR (v.3.38.4) and clusterProfiler (v.4.10.0) R packages were used, respectively. A more detailed description of the RNA-seq data analysis pipeline is reported in the Supplementary Files (see RNA-seq data analysis).

### 4.13. Confirmatory Assays (qPCR, Flow Cytometry, Immunoblotting)

For qPCR confirmation studies, C2, NI-1, Cf2Th, and MDCK cells were seeded, exposed to SCE17, and total RNA was extracted as previously reported (see Incubation of cells for gene expression analysis and confirmatory assays and RNA extraction, respectively). Based on RNA-seq results obtained with C2 cells exposed to SCE17, the expression levels of DEGs related to cell cycle (*PLK1*, *CCNB2*, *CDKN1A,* and *TP53INP1*), DNA damage (*RAD51*), tumor microenvironment modulation (*CXCL13*), cancer-related pathways (*FOS* and *JUNB*), and cholesterol synthesis (*SQLE*) were determined by using qPCR assays. Details about reverse transcription and qPCR analysis, including primer sequences and main features (i.e., slope, efficiency, linear regression coefficients, and linear dynamic range) of each qPCR assay, are reported in the Supplementary Files (see Reverse transcription and qPCR analysis) and Table S8, respectively.

Concerning flow cytometry and immunoblotting confirmatory assays, C2 cells were seeded as described above (see Incubation of cells for gene expression analysis and confirmatory assays) and incubated for 24 and 48 h with two concentrations of SCE, i.e., SCE17 and SCE25. For apoptosis analysis, cells were collected, rinsed twice with PBS, and resuspended in binding buffer with AnnV-FITC (1 µL/100,000 cells). After 10 min of incubation at room temperature in the dark, PI (final concentration, 1 μg/mL) was added. After 1 min of incubation, flow cytometric analysis was performed using the Cytoflex flow cytometer (Beckman Coulter Life Sciences, Warsaw, Poland). In addition, to assess a possible involvement of CASP3/7, the CellEvent Caspase 3/7 Green Flow Cytometry Assay Kit (Thermo Fisher Scientific, Warsaw, Poland) was used following the manufacturer’s instructions.

As for cell cycle evaluation, cells were washed twice with PBS, fixed and permeabilized in ice-cold 70% ethanol, and incubated at 4 °C for at least two hrs. Then, FxCycle™ PI/RNase Staining Solution (Thermo Fisher Scientific, Warsaw, Poland) was used following the manufacturer’s instructions. The CytExpert 2.6 software (Beckman Coulter Life Sciences, Warsaw, Poland) was used for data analysis.

The protein expression levels of Bcl-2 and Bcl-XL, and γ-H2A.X were measured by immunoblotting. Details about the immunoblotting protocol, including the specific characteristics and dilutions of the antibodies used, are reported in the Supplementary Files (see Immunoblotting) and Table S9.

### 4.14. Statistical Analysis

Statistical analyses were performed using GraphPad Prism Software (Version 10.5.0, San Diego, CA, USA). Dose–response curves were generated using a non-linear regression [log(inhibitor) versus (vs.) normalized response, variable slope]. The IC_50_ and the R^2^ were provided by the software. As to qPCR results, the statistical analysis was performed applying the Mann–Whitney test (SCE17 vs. CTRL), while the Kruskal–Wallis test, followed by Dunn’s multiple comparison test, was used for cell cycle, AnnV/PI, CASP3/7. For immunoblotting analysis, a one-sample *t*-test was applied. For the validation of RNA-seq results, the Spearman correlation analysis was considered.

## 5. Conclusions

In the present study, the extract from the red alga SC was proved to be cytotoxic in canine MCT cell lines (25 μg/mL < IC_50_ < 35 μg/mL); however, the observed SI value (<2.0) would suggest SCE partially affects normal canine cell lines, too (MDCK and Cf2Th). The potential anti-cancer activity of SCE was confirmed by the overall transcriptional inhibition of MELPATH, cell cycle arrest, apoptosis, and DNA damage. Chemical analysis of SCE would suggest these effects would be primarily due to the presence of the triterpene BET.

Overall, the presented results confirm SCE as a potential source of anti-cancer derivatives and demonstrate how NCs and green pharmacology represent a promising strategy in veterinary and comparative oncology. Nevertheless, additional, comparative, and multidisciplinary studies (e.g., chemical, molecular, -omic), encompassing both human and canine cancer and normal cell lines, are needed. These would help to deepen our understanding of present (SCE) and perspective (NCs) unsolved issues and potential interpretative biases. Such research would reinforce the “One Health, One Medicine” paradigm, enabling a more rapid development of novel therapies while enhancing our comparative knowledge and understanding of cancer biology.

## Data Availability

Raw Illumina sequencing data have been deposited in GenBank (SRA) under the BioProject accession PRJNA1287075.

## Supplementary Materials

The following supporting information can be downloaded at: https://www.mdpi.com/article/10.3390/md23120457/s1, Supplementary Files containing Supplementary Methods and Results; Table S1. SCE cytotoxicity in canine cancer vs. normal cell lines; Table S2. SCE selectivity index (SI); Table S3. Chemical composition of the dichloromethane/methanol SCE; Table S4. Sequencing and mapping results of C2 cells exposed to SCE8 and SCE17 for 48 h; Table S5. Differentially expressed genes; Table S6. GOandKEGGenrichmentanalysis; Table S7. Concentrations of algal extracts used for the preliminary cytotoxicity screening; Table S8. Oligonucleotide primer sequences and qPCR assay parameters; Table S9 Antibodies used for immunoblotting analyses; Figure S1. Algae extracts cytotoxicity; Figure S2. ATR-FTIR analysis of SC organic extract; Figure S3. Mass spectrum of BET; Figure S4. Expression profile of DEGs in common between SCE8 vs. CTRL and SCE17 vs. CTRL comparisons; Figure S5. Dot plot of the 20 GO terms most significantly enriched by up-regulated DEGs obtained from the SCE17 vs. CTRL comparison; Figure S6. Dot plot of KEGG enrichment analysis of up-regulated DEGs found in the SCE17 vs. CTRL comparison; Figure S7. Correlation analysis of RNA-seq and qPCR mRNA levels; Figure S8. qPCR results of target genes related to cell cycle and DNA damage; Figure S9. qPCR results of target genes associated with cancer-related pathways, cholesterol synthesis, and tumor microenvironment modulation; Figure S10. Percentage of SubG0 cells [69,70,71,72,73,74,75,76,77,78,79,80,81,82,83].

## Abbreviations

The following abbreviations are used in this manuscript:

AB	Alamar Blue
A/G	Alanine-Glutamine
AnnV	Annexin V
AnnV/PI	Annexin V/Propidium iodide
*AOAH*	Acyloxyacyl Hydrolase
ATR_FTIR	Attenuated Total Reflection Fourier Transform Infrared Spectroscopy
Bcl-2	B-cell lymphoma
Bcl-XL	B-cell lymphoma extra large
BET	Betulin
BH	Benjamini–Hochberg
BSA	Bovine serum albumin
CASP3/7	Caspase 3/7
*CCNB2*	Cyclin B2
*CDKN1A*	Cyclin Dependent Kinase Inhibitor 1A
*CPT1A*	Carnitine Palmitoyltransferase 1A
CTRL	Control
*CXCL13*	CXC Motif Chemokine Ligand 13
DAD	Diode array
ΔΔCT	Comparative Cycle Threshold
DEGs	Differentially expressed genes
DGE	Differential Gene Expression
DMEM	Dulbecco’s Modified Eagle Medium
DMSO	Dimethyl sulphoxide
EDTA	Ethylenediaminetetraacetic acid
FAME	Fatty acid methyl esters
FBS	Fetal bovine serum
fc	Fold changes
*FOS*	*FOS* proto-oncogene
GC/MS	Gas chromatography coupled to mass spectrometry
GO	Gene Ontology
H-ESI	Heated electrospray ionization
*HK2*	Hexokinase 2
IC_50_	Half maximal inhibitory concentration
*LAMC2*	Laminin Subunit Gamma 2
*JUNB*	JUNB proto-oncogene
KEGG	Kyoto Encyclopedia of Genes and Genomes
MCT	Mast cell tumor
MDCK	Madin-Darby canine kidney
MELPATH	Mevalonate pathway
MEM	Minimum Essential Medium
NCs	Natural compounds
NEAA	Non-essential amino acids
NIST	National Institute of Standards and Technology
NRU	Neutral Red Uptake
PBS	Phosphate-buffered saline
PI	Propidium iodide
P/S	Penicillin-streptomycin
PYR	Sodium pyruvate
qPCR	Quantitative RT-PCR
*RAD51*	RAD51 Recombinase
RIN	RNA Integrity Number
RPMI	Roswell Park Memorial Institute
Rt	Retention times
*PIM2*	Pim-2 Proto-Oncogene
*PLK1*	Polo-like kinase 1
SDS	Sodium dodecyl sulphate
SC	*Sphaerococcus coronopifolius*
SCE	*Sphaerococcus coronopifolius* extract
SCE8	*Sphaerococcus coronopifolius* extract, 8.33 µg/mL
SCE17	*Sphaerococcus coronopifolius* extract, 16.66 µg/mL
SCE25	*Sphaerococcus coronopifolius* extract, 25.00 µg/mL
SI	selectivity index
*SQLE*	Squalene Epoxidase
SRB	Sulforhodamine B
TBST	Tris-buffered saline with Tween 20
TMM	Trimmed mean of M-values
*TP53INP1*	Tumor Protein 53 Inducible Nuclear Protein 1
