# Supplementary material for: Anti-Cancer Activity of Sphaerococcus coronopifolius Algal Extract: Hopes and Fears of a Possible Alternative Treatment for Canine Mast Cell Tumor
Source: Mar Drugs. 2025 Nov 28;23(12):457. doi: 10.3390/md23120457 (PMC12735324; doi:10.3390/md23120457)
Supplement: Supplementary file 1 [file marinedrugs-23-00457-s001.zip › SupplementaryFiguresTables_marinedrugs3969498.pdf]

Supplementary Tables and Figures

Anti-cancer activity of *Sphaerococcus coronopifolius* Algal Extract: Hopes and Fears of A Possible Alternative Treatment for Canine Mast Cell Tumor

Greta Mucignat[1,†], Fatima Lakhdar[2,†], Hanane Maghrebi[1], Ewa Dejnaka[3], Lorena Lucatello[1], Bouchra Benhniya[2], Francesca Capolongo[1], Samira Etahiri[2], Marianna Pauletto[1], Aleksandra Pawlak[3,4,5], Mery Giantin[1,*], Mauro Dacasto[1,*]

[1] Department of Comparative Biomedicine and Food Science, University of Padua, I-35020 Agripolis Legnaro, Padua, Italy.
[2] Laboratory of Marine Biotechnology and Environment, Department of Biology, CNRST Labelled Research Unit, Faculty of Sciences, Chouaib Doukkali University, 24000 El Jadida, Morocco.
[3] Department of Pharmacology and Toxicology, Faculty of Veterinary Medicine, Wrocław University of Environmental and Life Sciences, 50-375 Wrocław, Poland.
[4] Department of Physiology and Pharmacology, University of Georgia, Athens, GA 30602, USA.
[5] SMART Pharmacology, Precision One Health Initiative, University of Georgia, Athens, GA 30602, USA.
* Correspondence: mery.giantin@unipd.it, mauro.dacasto@unipd.it
†These authors have contributed equally to this work

Supplementary Tables pages 2-9

Supplementary Figures pages 10-18

Supplementary Tables

Table S1. SCE cytotoxicity in canine tumor vs. normal cell lines. IC_{50} and R^2 values obtained with AB assay in four canine cell lines: C2 and NI-1 (MCT), Cf2Th and MDCK (normal). Each curve is based on three independent biological replicates, with each condition tested in sextuplicate.

**Cell line**	**Cytotoxicity (AB assay)**	
	**IC_{50}**	**R^2**
**C2**	26.41 µg/mL	0.97
**NI-1**	28.82 µg/mL	0.98
**Cf2Th**	43.90 µg/mL	0.98
**MDCK**	34.25 µg/mL	0.96

IC_{50}, half maximal inhibitory concentration; MDCK, Madin-Darby canine kidney; MCT, mast cell tumor; SCE, *Sphaerococcus coronopifolius* extract; SI, selectivity index

Table S2. SCE selectivity index (SI). The index was obtained by comparing IC_{50} values of canine tumor (C2 and NI-1) vs. normal (Cf2Th and MDCK) cell lines (i.e. IC_{50} of normal cells/IC_{50} of tumor cells ratio)

**Cell line**	**Selectivity Index (SI)**	
	**Cf2Th**	**MDCK**
**C2**	1.66	1.29
**NI-1**	1.52	1.19

IC_{50}, half maximal inhibitory concentration; SCE, *Sphaerococcus coronopifolius* extract; SI, selectivity index

Table S3. Chemical composition of the dichloromethane/methanol SCE

Peak	Rt	m/z	Fragment peaks	Compound name a	Class	Formula	Library or database used for the identification
**1**	14.18	242.3	74.1 (100), 143.2, 199.2, 111, 213	Tetradecanoic acid, methyl ester	Fatty acid	$C_{15}H_{30}O_2$	NIST WebBook NIH MASS SPECTRAL LIBRARY
**2**	18.20	256.4	74.1 (100), 143.2, 157, 87, 199	Pentadecanoic acid, methyl ester	Fatty acid	$C_{16}H_{32}O_2$	NIST WebBook NIH MASS SPECTRAL LIBRARY
**3**	20.58	182.1	81 (100), 67, 95, 122	Undec-10-ynoic acid	Fatty acid	$C_{11}H_{18}O_2$	NIST WebBook MassBank
**4**	20.65	296.2	73 (100), 75, 81, 97, 143, 123	Phytol	Diterpene	$C_{20}H_{40}O_2$	NIST WebBook MassBank
**5**	20.95	298.3	74.1 (100), 87, 134.2, 199.2	Octadecanoic acid, methyl ester	Fatty acid	$C_{19}H_{38}O_2$	NIST WebBook MassBank
**6**	21.10	204.3	91.1 (100), 79, 93.1, 133	β-Caryophyllene	Sesquiterpene	$C_{15}H_{24}$	NIST WebBook NIH MASS SPECTRAL LIBRARY
**7**	21.66	222.4	81.1 (100), 95, 109, 123, 151, 180	8-Hexadecyne	Alcan	$C_{16}H_{30}$	NIST WebBook NIH MASS SPECTRAL LIBRARY
**8**	22.87	155.1	109 (100), 83, 125	1-Fluoro-2-methyl-4-nitrobenzene	Halogenated aromatic compound	$C_7H_6FNO_2$	MassBank NIH MASS SPECTRAL LIBRARY
**9**	24.03	164.2	149 (100), 121.1, 91, 77	2-(1,1-dimethylethyl)-5-methyl Phenol	Phenolic compound	$C_{11}H_{16}O$	NIST WebBook
**10**	24.47	314.4	155 (100), 74, 84, 98	Hexadecanedioic acid, dimethyl ester	Fatty acid	$C_{18}H_{34}O_4$	NIST WebBook NIH MASS SPECTRAL LIBRARY
**11**	24.66	296.4	55 (100), 74, 69, 83, 111, 222	(Z)-9- Octadecenoic acid, methyl ester	Fatty acid	$C_{19}H_{36}O_2$	NIST WebBook

**12**	24.88	442.7	95 (100), 69, 81, 203, 147.1, 189, 107, 203.2, 257.2, 302	BET	Triterpene	C30H50O2	NIST WebBook NIH MASS SPECTRAL LIBRARY
**13**	25.33	384.6	271 (100), 272, 69, 356	Desmosterol	Steroid	C27H44O	NIST WebBook NIH MASS SPECTRAL LIBRARY
**14**	25.39	254.2	255.2 (100), 77, 107, 131, 95	Anisole, 2-sec-butyl-4,6-dinitro	organic nitroaromatic compound	C11H14N2O5	NIST WebBook
**15**	25.46	410.7	69 (100), 83, 139, 203, 303	Squalene	Triterpene	C30H50	NIST WebBook NIH MASS SPECTRAL LIBRARY
**16**	25.73	394.2	177 (100), 179	Luzonenone	Sesquiterpene	C15H22Br2O2	NIH MASS SPECTRAL LIBRARY
**17**	26.26	412.6	255 (100), 159.2, 83, 97, 147	Stigmasterol	Steroid	C29H48O	NIST WebBook NIH MASS SPECTRAL LIBRARY

BET, betulin; Rt, Retention time (min); SCE, *Sphaerococcus coronopifolius* extract.
[a] Compounds listed in order of retention time.

Table S4. Sequencing and mapping results of C2 cells exposed to SCE8 and SCE17 for 48 hrs

RNA-seq library	Nr. raw reads	Nr. reads survived after trimming	Nr. reads pseudo-aligned
C2_CTRL_1	20,979,499	20,591,693	16,928,571
C2_CTRL_2	24,865,458	24,362,084	19,931,311
C2_CTRL_3	22,888,724	22,374,059	18,200,702
C2_CTRL_4	21,967,099	21,616,735	17,835,794
C2_SCE8_1	22,314,319	21,985,386	17,731,015
C2_SCE8_2	22,176,918	21,698,094	17,845,776
C2_SCE8_3	21,968,002	21,576,641	17,562,044
C2_SCE8_4	23,519,948	23,033,718	18,871,457
C2_SCE17_1	22,982,071	22,595,180	17,997,138
C2_SCE17_2	25,100,817	24,630,155	20,034,619
C2_SCE17_3	22,455,068	22,097,171	18,013,866
C2_SCE17_4	22,672,162	22,184,658	18,079,623
**Mean**	22,824,174	22,395,464	18,252,660

CTRL, control; SCE8, *Sphaerococcus coronopifolius* extract, 8.33 μg/mL; SCE17, *Sphaerococcus coronopifolius* extract, 16.66 μg/mL

Table S7. Concentrations of algal extracts used for the preliminary cytotoxicity screening

Dose level	*Sphaerococcus coronopifolius* (μg/mL)	*Halopitys incurvus* (μg/mL)	*Laminaria ochroleuca* (μg/mL)
1	166.67	1330.00	700.00
2	83.33	1000.00	600.00
3	41.66	666.67	500.00
4	25.00	333.33	400.00
5	16.67	166.67	300.00
6	8.33	83.33	200.00
7	4.16	20.83	--

Table S8. Oligonucleotide primer sequences and qPCR assay parameters

Genes	Primers sequences	Slope	Efficiency	Error	Dynamic range	Reference
HPRT1	Fwd: TGCTCGAGATGTGATGAAGG<br>Rev: TCCCCTGTTGACTGGTCATT	-3.424	1.959	0.011	20.35 - 31.52	[81]
CGI-119	Fwd: TCTACAATCTAAGAGAGATTTCAGCAA<br>Rev: TTCCTGACAAGCACAAAATCC	-3.298	2.01	0.024	20.05 - 31.73	[82]
CCZ1	Fwd: TGAAGCACTGCATTTAATTGTTTAT<br>Rev: CTTCGGCAAAAATCCAATGT	-3.525	1.922	0.034	22.17 - 33.02	[83]
RAD51	Fwd: GGCCATGTACATTGACACTGA<br>Rev: CACTGCCAGAGAGGCCATA	-3.154	2.075	0.009	22.48 - 34.72	[81]
CCNB2	Fwd: CCAGTACAGATGGAAATGTTG<br>Rev: AGGTTCTCTTCCTTCATGGAGAT	-3.284	2.016	0.009	21.79 - 32.81	[81]
PLK1	Fwd: TGAGCAAGAAAGGGCACAGT<br>Rev: AGGCAAGAGGTCTCAAAAGGT	-3.316	2.003	0.006	20.03 - 31.61	Designed *ex novo*
CXCL13	Fwd: CCATCCAGCTCATTGAAAGGC<br>Rev: TGGGAGGGTTCAAGCATACAA	-3.278	2.019	0.044	26.84 - 34.45	Designed *ex novo*
SQLE	Fwd: ACTTGTTGACATCCGAGGAGAA<br>Rev: TCCAGGAACGGTTCTTTGAGG	-3.365	1.982	0.024	20.12 - 31.44	Designed *ex novo*
TP53INP1	Fwd: TTGTTGACTTCATAGACACT<br>Rev: CCAAGCACTCCAGAGACGC	-3.604	1.894	0.044	25.21 - 35.00	Designed *ex novo*
FOS	Fwd: TCCTACTACCACTCACCGGC<br>Rev: CGTCGGGATGAAGTTGGCA	-3.421	1.96	0.007	20.35 - 31.56	Designed *ex novo*
JUNB	Fwd: AACAGCCCTTCTACCACGAC<br>Rev: GGGGCTTTGAGACTTCGGTA	-3.452	1.948	0.022	22.23 - 30.23	Designed *ex novo*
CDKN1A	Fwd: GGACCTGTCGCTGACCTG<br>Rev: GGCGTTTGGAGTGATAGAAA	-3.521	1.923	0.009	24.01 - 35.0	Designed *ex novo*

HPRT1, Hypoxanthine Phosphoribosyltransferase 1; *CGI-119*, Comparative Gene Identification-119; *CCZ1*, CCZ1 Homolog, Vacuolar Protein Trafficking and Biogenesis Associated; *RAD51*, RAD51 Recombinase; *CCNB2*, Cyclin B2; *PLK1*, Polo-like kinase 1; *CXCL13*, CXC Motif Chemokine Ligand 13; *SQLE*, Squalene Epoxidase; *TP53INP1*, Tumor Protein 53 Inducible Nuclear Protein 1; *CDKN1A*, Cyclin Dependent Kinase Inhibitor 1A. qPCR: quantitative RT-PCR.

Table S9. Antibodies used for immunoblotting analyses

Primary Antibody	ID	Host species	Clonality	Dilution	Secondary Antibody (dilution)
anti-γ-H2A.X clone 9F3	ab26350	Mouse	monoclonal	1:1000	Goat Anti-Mouse Immunoglobulins/HRP (P0447, 1:20,000 concentration in TBST solution)
anti-β-actin clone C4	sc-47778	Mouse	monoclonal	1:2000	
anti-Bcl-2	sc-7382	Mouse	monoclonal	1:1000	
anti-Bcl-XL (54H6)	2764	Rabbit	monoclonal	1:1000	Goat Anti-Rabbit Immunoglobulins/HRP (P0448, 1:10,000 concentration in TBST solution)

Bcl-2, B-cell lymphoma 2; Bcl-XL, B-cell lymphoma-extra-large; HRP, horseradish peroxidase; TBST, Tris-buffered saline with Tween 20.

Supplementary Figures

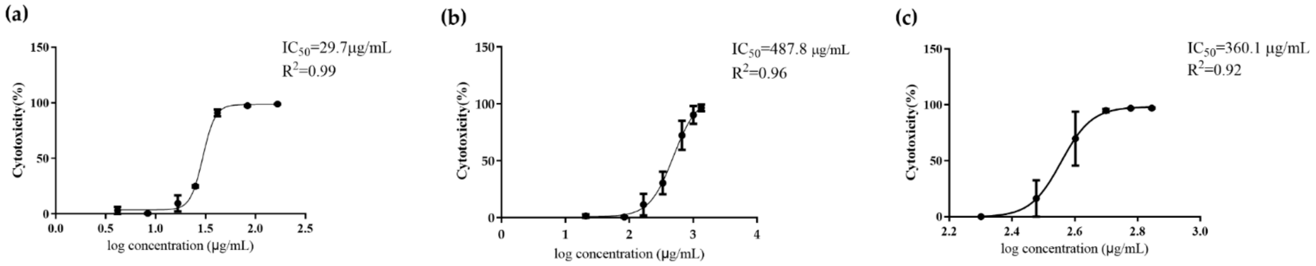

Figure S1. Algae extracts cytotoxicity. Dose-response curves, half maximal inhibitory concentration (IC_{50}) and R^2 values obtained in the C2 cell line using the Alamar blue (AB) assay. Candidate algae extracts were from *Sphaerococcus coronopifolius* (**a**), *Halopitys incurvus* (**b**), and *Laminaria ochroleuca* (**c**). Each curve is based on two independent biological replicates, with each condition tested in sextuplicate.

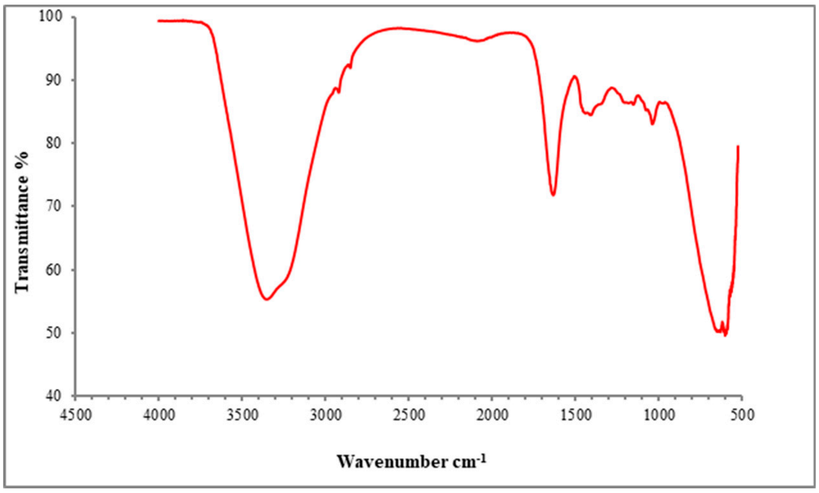

Figure S2. ATR-FTIR analysis of *Sphaerococcus coronopifolius* organic extract.
ATR-FTIR, Attenuated Total Reflection Fourier Transform Infrared Spectroscopy.

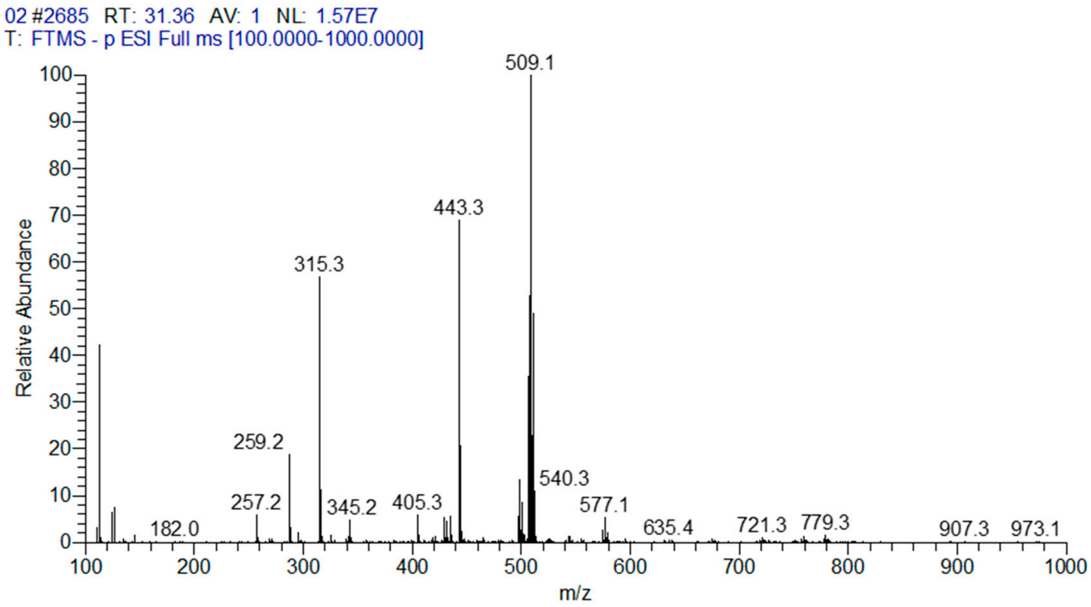

Figure S3. Mass spectrum of betulin (BET). The molecular ion [M-H] $^{+}$ characteristic of BET at m/z = 443.3 is shown.

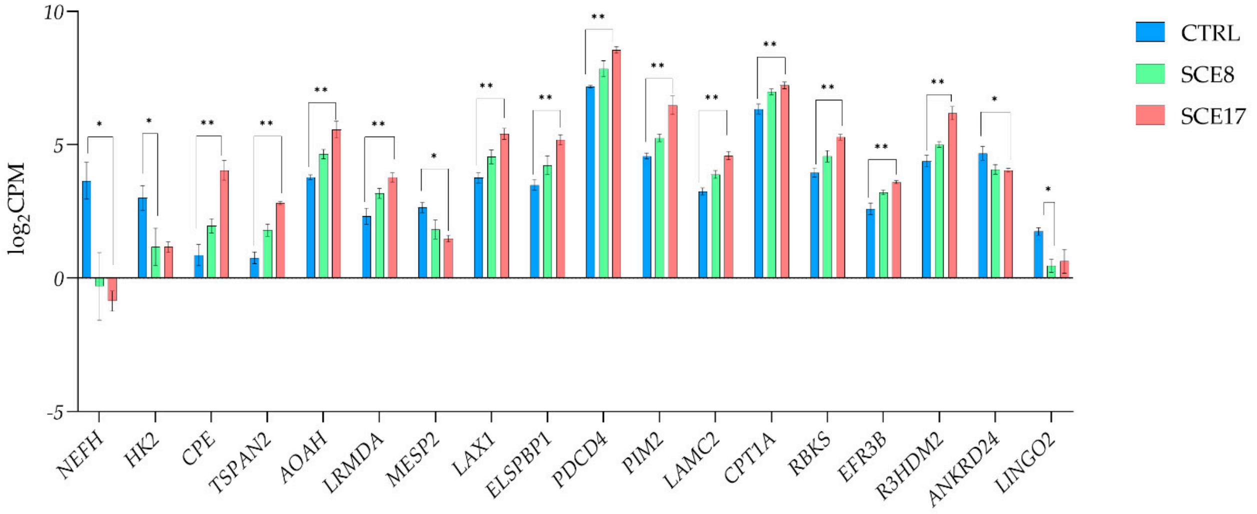

Figure S4. Expression profile of DEGs in common between SCE8 vs. CTRL and SCE17 vs. CTRL comparisons. For each gene, the expression levels were reported as log2 counts per million (CPM). Reads were previously filtered and TMM normalized. Statistical analysis: Kruskal-Wallis test followed by Dunn's multiple comparison test. *: $p < 0.05$. **: $p < 0.01$. SCE8, *Sphaerococcus coronopifolius* extract, 8.33 μg/mL; SCE17, *Sphaerococcus coronopifolius* extract, 16.66 μg/mL; TMM, trimmed mean of M-values

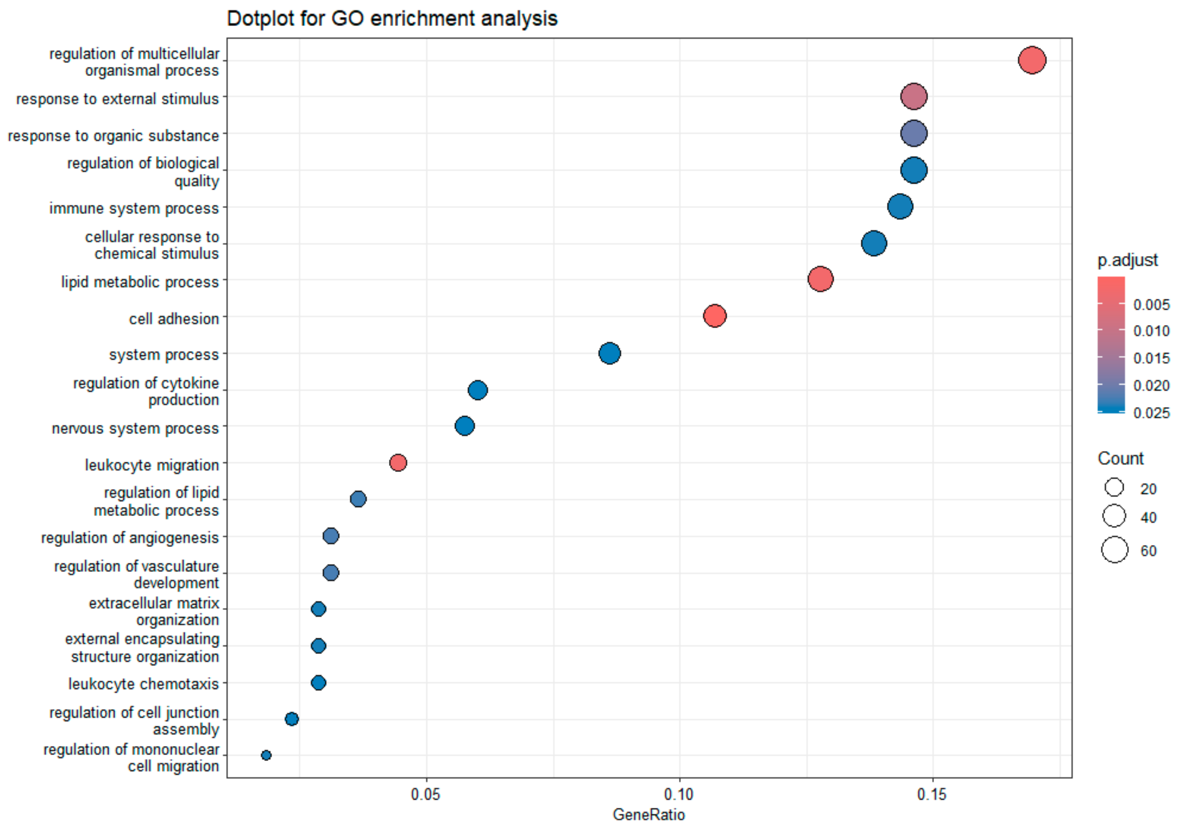

Figure S5. Dot plot of the 20 GO terms most significantly enriched by up-regulated DEGs obtained from the SCE17 vs. CTRL comparison. The dot size represents the number of genes belonging to each pathway. The color gradient is related to the level of significance, adjusted with the BH method.BH, Benjamini-Hochberg; CTRL, control; DEGs, Differentially Expressed Genes; GO, Gene Ontology; SCE17, *Sphaerococcus coronopifolius* extract, 16.66 µg/mL

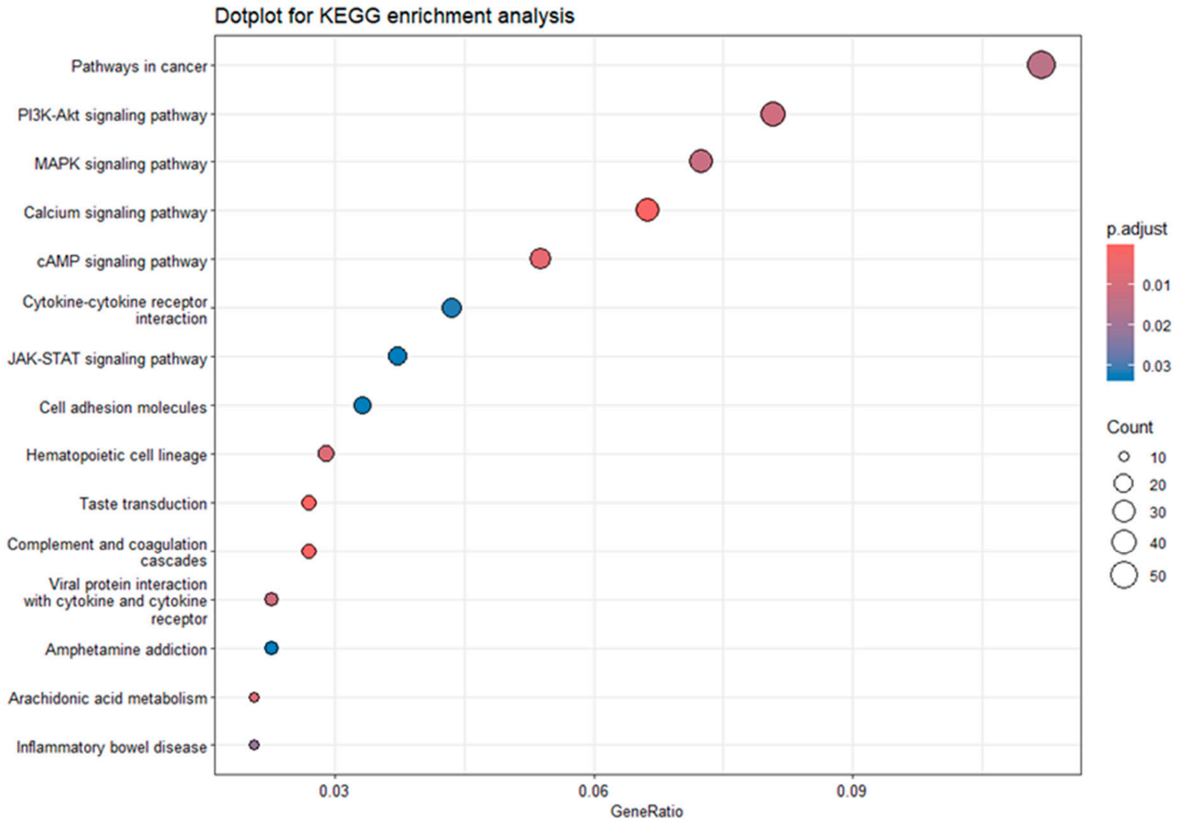

Figure S6. Dot plot of KEGG enrichment analysis of up-regulated DEGs found in the SCE17 vs. CTRL comparison. The dot size represents the number of genes belonging to each pathway. The color gradient is related to the level of significance, adjusted with the BH method.BH, Benjamini-Hochberg; CTRL, control; DEGs, Differentially Expressed Genes; KEGG, Kyoto Encyclopedia of Genes and Genomes; SCE17, *Sphaerococcus coronopifolius* extract, 16.66 μg/mL

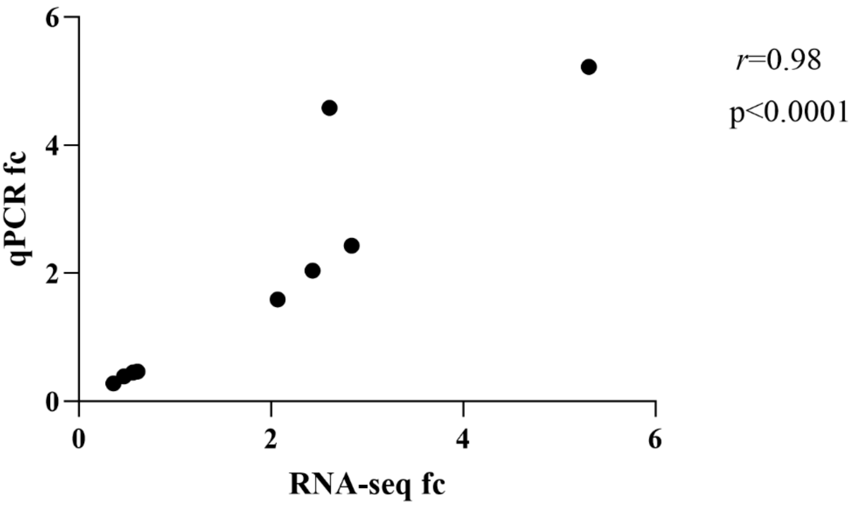

Figure S7. Correlation analysis of RNA-seq and qPCR mRNA levels. The dot plot shows the Spearman's correlation coefficient (*r*) and the resulting level of significance obtained when comparing the RNA-seq and qPCR mRNA levels (in terms of fc) of 9 target DEGs identified following the RNA-seq analysis. DEGs, differentially expressed genes; fc, fold changes; qPCR, quantitative RT-PCR.

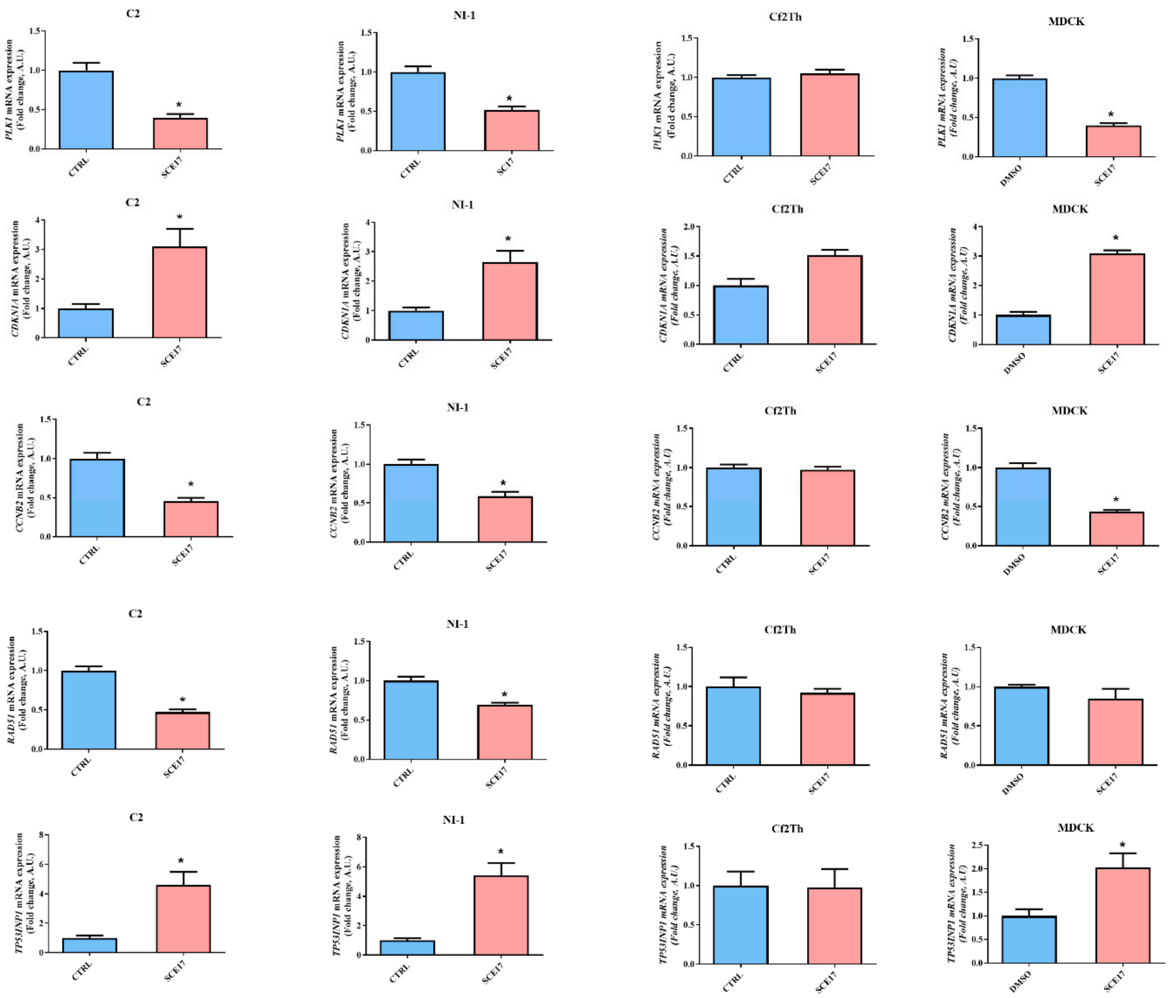

Figure S8. qPCR results of target genes related to cell cycle and DNA damage. C2, NI-1, CF2TH and MDCK cells were exposed for 48 hrs either to SCE17 or 0.33% DMSO (control cells). *CCNB2*, Cyclin B2; *CDKN1A*, Cyclin Dependent Kinase Inhibitor 1A; CTRL, control; DMSO, dimethyl sulfoxide; *PLK1*, Polo-like kinase 1; qPCR, quantitative RT-PCR; *RAD51*, RAD51 Recombinase; SCE17, *Sphaerococcus coronopifolius* extract, 16.66 µg/mL; *TP53INP1*, Tumor Protein 53 Inducible Nuclear Protein 1.
*: $p < 0.05$.

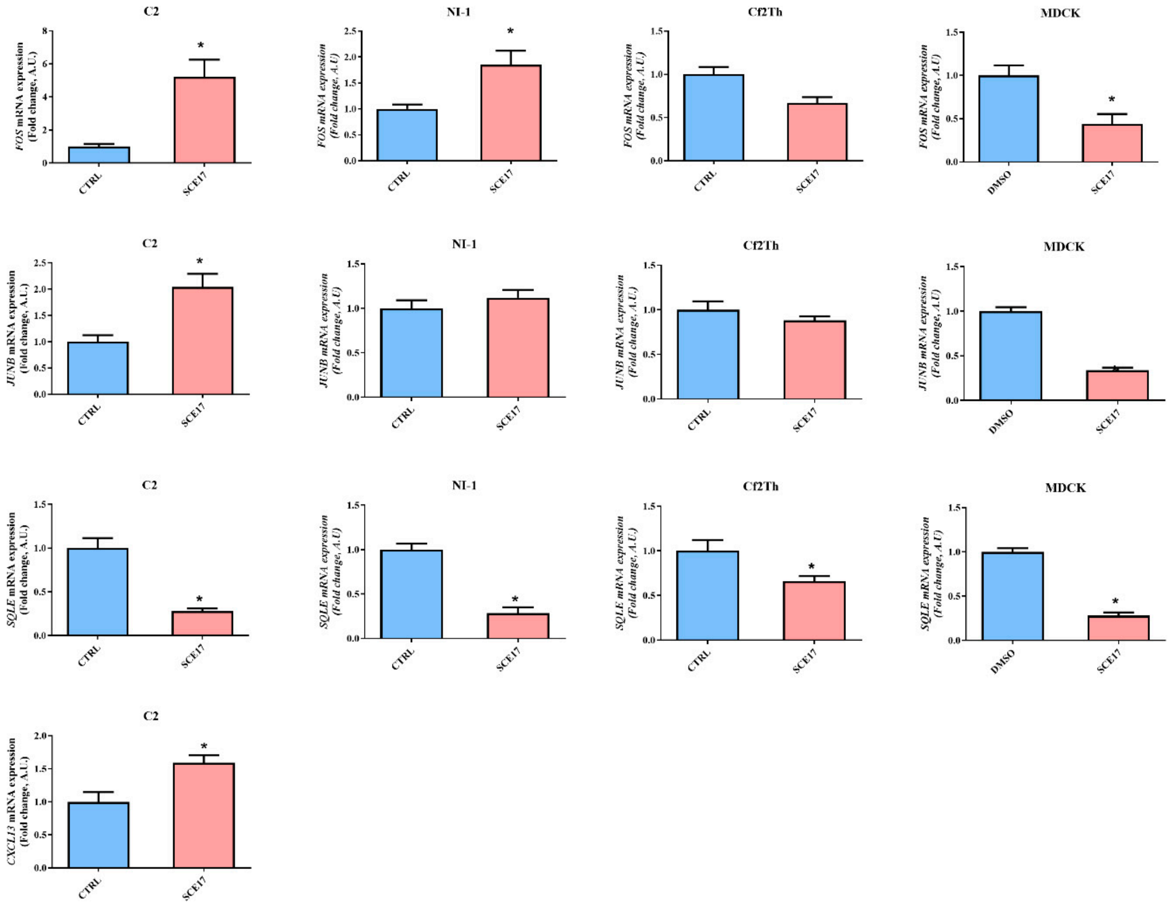

Figure S9. qPCR results of target genes associated with cancer-related pathways, cholesterol synthesis and tumor microenvironment modulation. C2, NI-1, CF2TH and MDCK cells were exposed for 48 hrs either to SCE17 or 0.33% DMSO (control cells). *CXCL13*, *CXC Motif Chemokine Ligand 13;* CTRL, control; DMSO, dimethyl sulfoxide; *FOS*, *FOS* proto-oncogene; *JUNB*, *JUNB*, proto-oncogene; qPCR, quantitative RT-PCR; SCE17, *Sphaerococcus coronopifolius* extract, 16.66 µg/mL; *SQLE*, Squalene Epoxidase.
*: $p < 0.05$.

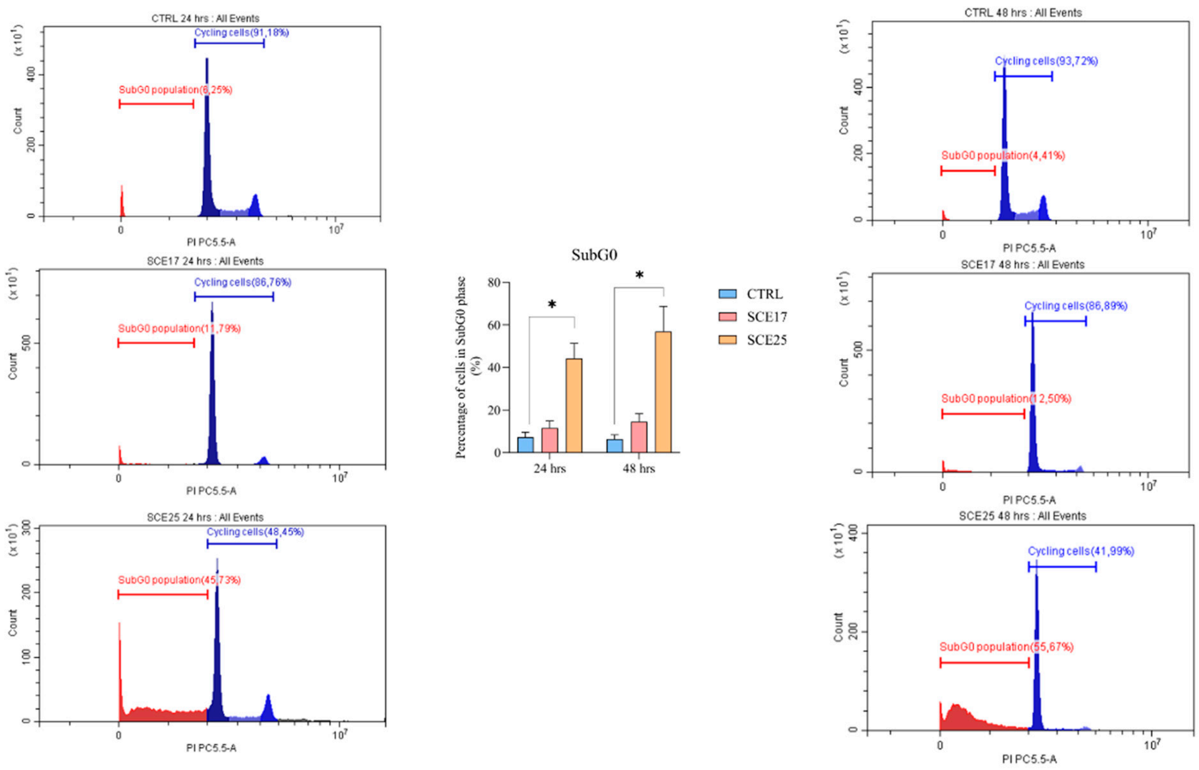

Figure S10. Percentage of SubG0 cells. C2 cells were exposed to SCE17 and SCE25 for 24 and 48 hrs. The percentage of SubG0 cells is reported for each condition tested. Graphs reporting the statistical analysis are shown alongside explanatory histograms. For all experiments, three biological replicates were used per condition. Statistical analysis: Kruskal-Wallis test followed by Dunn's multiple comparison test. *: $p < 0.05$. CTRL, control; SCE17, *Sphaerococcus coronopifolius* extract, 16.66 μg/mL; SCE25, *Sphaerococcus coronopifolius* extract, 25 μg/mL.
