# Supplementary material for: Anti-Cancer Activity of Sphaerococcus coronopifolius Algal Extract: Hopes and Fears of a Possible Alternative Treatment for Canine Mast Cell Tumor
Source: Mar Drugs. 2025 Nov 28;23(12):457. doi: 10.3390/md23120457 (PMC12735324; doi:10.3390/md23120457)
Supplement: Supplementary file 1 [file marinedrugs-23-00457-s001.zip › SupplementaryFiles_marinedrugs3969498.pdf]

Supplementary Files

Anti-cancer activity of *Sphaerococcus coronopifolius* Algal Extract: Hopes and Fears of A Possible Alternative Treatment for Canine Mast Cell Tumor

Greta Mucignat[1,+], Fatima Lakhdar[2,+], Hanane Maghrebi[1], Ewa Dejnaka[3], Lorena Lucatello[1], Bouchra Benhniya[2], Francesca Capolongo[1], Samira Etahiri[2], Marianna Pauletto[1], Aleksandra Pawlak[3,4,5], Mery Giantin[1,*], Mauro Dacasto[1,*]

1 Department of Comparative Biomedicine and Food Science, University of Padua, 35020 Agripolis Legnaro, Padua, Italy.
2 Laboratory of Marine Biotechnology and Environment, Department of Biology, CNRST Labelled Research Unit, Faculty of Sciences, Chouaib Doukkali University, 24000 El Jadida, Morocco.
3 Department of Pharmacology and Toxicology, Faculty of Veterinary Medicine, Wrocław University of Environmental and Life Sciences, 50-375 Wrocław, Poland.
4 Department of Physiology and Pharmacology, University of Georgia, Athens, GA 30602, USA.
5 SMART Pharmacology, Precision One Health Initiative, University of Georgia, Athens, GA 30602, USA.
* Correspondence: mery.giantin@unipd.it, mauro.dacasto@unipd.it
[+]These authors have contributed equally to this work

Supplementary Results pages 2-3

Supplementary Materials and Methods pages 4-9

Supplementary Results

Chemical characterization of SCE

The organic SCE was analyzed using ATR-FTIR spectroscopy in the range of 500–4500 cm−1, and the FTIR spectra in Figure S2 show the evolution of the absorption bands of the organic SCE. The broad spectral bands located in the region between 3200 and 3314 cm-1 were attributed to the stretching of the OH group related to carbohydrates, proteins, or polyphenols. Peaks obtained at 2935 cm-1 and 2838 cm-1 were attributed to the vibration of the CH2 and CH aliphatic groups of lipids. A band in the region from 2050 to 2100 cm-1 was associated to the −C≡C−, which corresponded to the alkynes group, probably deriving from flavonoids and terpenoids. Peaks at 1624 cm-1 corresponded to the C=O group of fatty acid esters or the N─H group of amines. Bands obtained in the region between 1436 to 1420 cm-1 were attributed to the CH2 scissoring from saturated lipids, and peaks obtained at 1393 cm-1 for organic SCE corresponded to the CH3 symmetric band from lipids and proteins. Another band located at 1320 cm-1 might be attributed to the C−N group of proteins or the deformation of the C−H group from Chlorophyll a. Bands between 1200 and 1027 cm-1, were attributed to the stretching of the C−O band of polysaccharides and the P=O groups of phospholipids [69]. Finally, bands located in the region from 590 to 620 cm-1 might be attributed to a halogenated compound. All these chemical bonds were favorable factors that indicate the abundance of active sites on the algal surface.

Mass spectra of the dichloromethane/methanol extract of SC were compared with those from reference samples and with mass spectra from a data library or the NIST database. The chemical analysis revealed a diverse array of phytochemical compounds. After transesterification of the crude algal extract, fatty acid constituents were determined using GC/MS analysis. Based on the retention times (Rt) and molecular weights inferred from the GC/MS chromatogram, further confirmed by HPLC/UV/MS analysis, seventeen compounds were identified in the SCE (Table S3). These compounds showed more than 80% similarity with the standard mass spectra in the library, representing approximately 70% of the relative area in this extract and exhibiting a chemotaxonomic profile similar to other algae species. The mass spectra showed a distinct molecular ion peak, observed at m/z=443.3[M+H]+, a fragment that supports the proposed molecular formula of the compound (Figure S3). The presence of the highest sharps peak with the Rt of 24.88 min and the highest area percentage has revealed a similar standard Rt value of BET. Given the obtained spectral data, the major compound present in the SC extract was hypothesized to be BET.

Confirmatory qPCR results of SCE selectivity against cancer cells

In the C2 cell line, qPCR investigations showed that *RAD51*, *CCNB2*, *PLK1*, and *SQLE* were significantly down-regulated, whereas *CDKN1A*, *TP53INP1*, *JUNB*, *FOS*, and *CXCL13* showed a significant up-regulation ($p < 0.05$ in both cases; Figures S8 and S9). As previously demonstrated (see also Figure S7), these qPCR results corroborate those obtained with transcriptome analysis. In the NI-1 cell line, the RNA-seq analysis was not performed; nevertheless, this MCT cell line showed target gene mRNA levels similar to those observed in C2 cells. Overall, this transcriptional consistency suggests that SCE targets similar cellular pathways in canine MCT cell lines. However, qPCR results from non-cancerous cell lines (Cf2Th and MDCK) indicated distinct responses to SCE: while MDCK data were sometimes comparable to those seen in MCT cell lines, Cf2Th cells showed minimal changes and/or opposite trends in target gene mRNA levels.

More specifically, in C2 and NI-1 cells, a significant down-regulation of *RAD51*, *CCNB2*, *PLK1*, and *SQLE* was observed, suggesting an inhibition of cell cycle progression and DNA repair mechanisms. Conversely, in Cf2Th and MDCK cells, such a down-regulation was less marked or absent (e.g., *PLK1* in Cf2Th). In addition, a significant up-regulation of *CDKN1A*, *JUNB*, *TP53INP1,* and *FOS*, more consistent in C2 compared to NI-1 cells, was observed. Noteworthy, *TP53INP1* and *FOS* were the most up-regulated genes. On the other hand, non-cancerous cells showed a minimal up- or down-regulation of these target genes, further suggesting a cell type specific response to SCE. Finally, a constitutive expression of the *CXCL13* gene was merely observed in C2 cells.

Supplementary methods

Preliminary screening of algal extracts cytotoxicity using AB assay

Canine C2 cells were seeded in 96-well flat-bottom plates at a density of 3 × 10^4 cells/well. Then, cells were exposed to increasing concentrations of the three algal extracts (see Table S5). After 48 hrs of incubation, 30 µL of AB reagent (0.15 mg/mL resazurin) was added to each well. Following an additional 3 hrs of incubation, the cell viability was measured by reading the fluorescence at 544 nm (excitation wavelength) and 590 nm (emission wavelength) and using a VICTOR™X4 Multilabel Plate Reader (Perkin Elmer, Waltham, USA). Negative controls, i.e., cells in culture with either medium alone or with the vehicle (DMSO, 0.33% final concentration), were included in each experiment. Two independent experiments were performed, and each concentration was tested in sextuplicate.

SCE cytotoxicity

A more robust confirmation of the best cytotoxic potential of SCE was made by using C2 cells and three different cytotoxicity tests, i.e., AB, SRB, and NRU assays. For AB and SRB, cells were seeded in 96-well flat-bottom plates at a density of 3×10^4 cells/well, while for NRU, 9×10^4 cells/well were used. Cells were exposed to the same SCE increasing concentrations reported in Table S1.

For the AB test, the same protocol previously described was used.

As to the SRB assay, after 48 hrs of incubation, cells were fixed with 50% cold trichloroacetic acid and incubated for 1 hr at 4°C, and then rinsed several times with water. After drying the plate at 37°C for 10 min, cells were stained with 100 μL/well of SRB dye solution (0.4%) and kept for 30 min at room temperature and in the dark. After washing with 1% acetic acid, the plate was dried as mentioned above and the protein-bound dye was dissolved in 200 μL of Tris base (10 mM, pH 10.5) solution. Finally, the optical density was measured at 570 nm by using the MULTISKAN™ GO Microplate Spectrophotometer (Thermo Fisher Scientific, Waltham, MA, USA).

With regard to NRU assay, after 48 hrs of incubation, cells were incubated for 3 hrs at 37°C and 5% of CO_2 with 150 μL/well of a neutral red stain solution (0.05 mg/mL). After incubation, cells were washed with 150 μL of phosphate-buffered saline (PBS) and, then, 150 μL/well of a de-stain solution (50% ethanol, 49% water, 1% acetic acid) was added. After 15 min of incubation, the optical density was read at 540 nm using MULTISKAN™ GO Microplate spectrophotometer. Even in this case, negative controls (i.e., cells with either medium alone or a final concentration of 0.33% DMSO) were included in each experiment. Three independent biological replicates, with each concentration tested in sextuplicate, were performed.

Chemical characterization of SCE

The ATR-FTIR analytical technique was employed to identify the main biochemical components present in SCE, including proteins, carbohydrates, lipids, and pigments. Lyophilized samples of SCE were finely ground in an agate mortar and analyzed by ATR-FTIR. Spectra were recorded between 4500 and 500 cm^{-1} using a NICOLET iS10 Thermo Fisher Scientific spectrometer (Madison, WI, USA) with a spectral resolution of 4 cm^{-1} and acquisition, which was done on 50 scans. The OMNIC program version 9 (Thermo Fisher Scientific, Waltham, MA, USA) was used for processing spectra and the resulting data. The recorded spectrum was the average of three replicates.

To characterize its chemical composition, the transesterified and air-dried SCE was analysed by using a (GC/MS) approach (GC TRACE 1300 TSQ 8000 evo system; Thermo Fisher Scientific, Waltham, MA, USA). Initially, an esterifying methanol-chloroform-hydrochloric methanol solution (300 μL-100 μL-500 μL) was prepared, with the hydrochloric methanol at 12% (*v*/*v*) concentration; therefore, 50 mg of dried SCEs were added to this solution, which was refluxed by heating at 80°C for 5 hrs. After cooling to room temperature, fatty acid methyl esters (FAME) were twice extracted by 500 μL of hexane at room temperature for 10 min. The resulting hexane solution was recovered and centrifuged for 5 min at 2000 rpm. After centrifugation, the supernatant hexane solution was collected using a microsyringe and dehydrated. One mg of the obtained extract was then solubilized in 1 mL of CH_2Cl_2 for total FAMEs GC/MS analysis. This latter was performed by injecting 1 μL of sample onto a TG-5 column (30 m × 0.25 mm × 0.25 μm) on the aforementioned GC/MS system. The GC was performed in the splitless mode, and for MS detection an electron ionization mode with an ionization energy of 70 eV was used. The maximum column temperature in the GC method was set to 300°C. The injector and interface temperature were held at 250°C, and helium was used as mobile phase, with a constant flow rate of 1 mL/min. The temperature program was started at 100°C and then increased to 180°C at a rate of 15°C min^{-1}, with a plateau for 5 min. The rate was then decreased to 5°C min^{-1} from 180 to 300°C, and this temperature was kept constant for 10 min. The ionization source temperature was 280°C with a mass range at m/z 40–500 in a 1 s cycle in a full scan acquisition mode. The identification of essential components was based on their mass spectra profiles and their comparison with those in the National Institute of Standards and Technology (NIST) Mass Spectral Library (Version 2017).

The organic SCE was redissolved and analyzed using a Thermo Scientific UltiMate 3000 HPLC coupled to a UV detector with diode array (DAD) and a Thermo Scientific™ Exactive™ electrospray ionization mass spectrometer (ESI-MS; Thermo Fisher Scientific, Waltham, MA, USA), operating in both positive and negative modes. Sample volumes of 20 μL were injected onto a C18 column (5 μm particle size; 150 mm × 4.6 mm, HyPURITY ADVANCE™). The mobile phase consisted of a gradient of 0.1% formic acid in water (A) and methanol (B). The gradient separation of the two solvents was carried out at a flow rate of 1 mL/min. The multistep gradient was as follows: 0–3 min: linear gradient from 5% to 25% B; 3–6 min: isocratic at 25% B; 6–9 min: linear gradient from 25% to 37% B; 9–13 min: isocratic at 37% B; 13–18 min: linear gradient from 37% to 54% B; 18–22 min: isocratic at 54% B; 22–26 min: linear gradient from 54% to 95% B; 26–29 min: isocratic at 95% B; 29–29.15 min: linear gradient back to initial conditions at 5% B; 29.15–36 min: isocratic at 5% B. UV-Vis detection was performed in the range of 200–400 nm, while the chromatogram was acquired at a wavelength of 280 nm. The TSQ-Endura triple quadrupole mass spectrometer (Thermo Fisher Scientific, San Jose, CA, USA) was used with heated electrospray ionization (H-ESI) as the ionization source in negative mode. The full scan range was set to 100-1000 m/z. Identification of components was carried out by comparing the absorption spectra with their m/z mass.

RNA-seq data analysis

Raw reads underwent quality control with FastQC software v.0.11.9 [70] and the amount of ribosomal RNA was checked using Bowtie2 v.2.2.9 [71] using an index built on SILVA databases of ribosomal small and large subunits for Bacteria, Archaea, and Eukaryotes (https://www.arbsilva.de/no_cache/download/archive/current/Exports/). Then, low-quality reads and adapters were removed using Trimmomatic v.0.36 [72]. Trimmed reads were pseudoaligned to the reference canine transcriptome (ROS_Cfam_1.0, Ensembl release 109) using Kallisto v.0.48.0 [73]. Transcripts were then imported into RStudio (R version 4.2.1) and collapsed to genes using the tximport package v.1.24.0 [74] and the annotations retrieved from Ensembl with R interface biomaRt v.2.54.0 [75]. The following steps of DGE analysis were carried out using the edgeR package v.3.38.4 [76]. First, genes with very low expression levels were removed (*filterByExpr*), and the remaining ones were normalized using the calcNormFactors function according to the trimmed mean of M-values (TMM). After fitting common and tagwise dispersion estimation (*estimateDisp*) and negative binomial generalized linear models (*glmQLFit*), differentially expressed genes (DEGs) were identified using quasi-likelihood F-test (*glmQLFTest*; [77]), setting the following contrasts: SCE8 vs. CTRL, SCE17 vs. CTRL. A Benjamini-Hochberg (BH) adjusted p-value < 0.05 and a $\log_2$ fold change (lfc) > 0.58 or < -0.58 were used to identify DEGs from each dataset. As to the SCE17 vs. CTRL comparison, up-regulated and down-regulated DEGs were submitted to over-representation analysis using Gene Ontology (GO: Biological Process) and Kyoto Encyclopedia of Genes and Genomes (KEGG) databases. To this purpose, *enrichGO* and *enrichKEGG* functions of clusterProfiler package v.4.10.0 [78] were applied.

Reverse transcription and qPCR analysis

First-strand cDNA synthesis from 1 μg total RNA was performed using the High-Capacity cDNA Reverse Transcription Kit (Thermo Fisher, Milan, Italy) following the manufacturer's instructions. cDNA (2.5 ng/reaction) was amplified and analyzed with qPCR Power SYBR Green PCR Master Mix (Applied Biosystems, Milan, Italy) using a Light Cycler 480 instrument (Roche Applied Science, Monza, Italy). For each candidate and reference gene, qPCR reactions were performed in two technical replicates and four biological replicates. Gene expression was quantified using the Comparative Cycle Threshold method (ΔΔCT; [79]). Results were presented as n-fold changes of the gene of interest in SCE-exposed cells relative to CTRL, with CTRL cells set as unity (1).

Immunoblotting

A total of 1.2 x 10^6 cells were rinsed with cold PBS, lysed with RIPA buffer (50 mM Tris-HCl, pH 7.5, 100 mM NaCl, 1% NP-40, protease inhibitors set), and incubated for 20 min on ice. After centrifuging at 10,000 rpm at 4°C for 12 min, sodium dodecyl sulphate (SDS) sample buffer was added to clear the supernatants, samples were then boiled at 95°C for 5 min. Immunoblotting was then performed following the steps reported in Pasaol et al. (2025) [80] with minor changes. To check for the efficiency of protein transfer and to perform total protein normalization, reversible membrane staining was done with Pierce™ Reversible Protein Stain Kit for Nitrocellulose Membranes (Thermo Fisher Scientific, Warsaw, Poland). Then, before blocking the membranes, Western Blot enhancer treatment was performed with Pierce™ Western Blot Signal Enhancer. Primary and secondary antibodies used are reported in Table S3. As regards quantitative analysis of images Image LabTM software (version 6.1.0; Bio-Rad Polska Sp. z o.o., Warsaw, Poland) was used, and the results were normalized to the total amount of protein in each lane (total protein normalization).
